# Supplementary material for: Functional and evolutionary synergy of trait components can explain the existence of leaf masquerade in katydids
Source: PLoS Biol. 2025 Nov 3;23(11):e3003468. doi: 10.1371/journal.pbio.3003468 (PMC12582443; doi:10.1371/journal.pbio.3003468)
Supplement: S1 Text — Fig A. Distributions of bird vision-calibrated normalized RGB reflectance values obtained from raw multispectral images of bramble leaves (top) and tree bark (bottom), which informed the color of the targets used in the field predation experiment. Vertical dashed blue lines in the bottom row indicate the mean RGB values of a random sample of recently thawed mealworms (Tenebrio molitor larvae), which were used as bait in the experiment and fall within the RGB distribution of the tree bark—the substrate upon which the targets were pinned. All data underlying this figure can be found in https://doi.org/10.5281/zenodo.14585161. Fig B. Survival plot for each color × shape treatment combination over time when exposed to wild avian predation (N = 1,296). Shading represents 95% confidence intervals for each treatment. All data underlying this figure can be found in https://doi.org/10.5281/zenodo.14585161. Fig C. Mean reflectance values for the tegmina of four randomly katydid species within a 290–700 nm spectral range (n = 2 per species). The inset plots these data in tetrachromatic avian color space using the blue tit (Cyanistes caeruleus) visual model, where points represent the photon catch of the blue tit shortwave (S), mediumwave (M), longwave (L), and ultraviolet (UV) cones. All data underlying this figure can be found in https://doi.org/10.5281/zenodo.14585161. Fig D. Estimated transitions from absence (gray nodes and tips) to presence (green nodes and tips) of green tegmen pigmentation, and vice versa (left), and patterns of mean tegmen aspect ratio (au) evolution (right) across a pruned molecular phylogeny of 58 sampled katydid species [23]. Pies at each node represent the posterior probability of the ancestor having each character state. All data underlying this figure can be found in https://doi.org/10.5281/zenodo.14585161. Fig E. All models included in each set of the phylogenetic path analysis with mean leafiness score as the response variable. The C-statistics [file pbio.3003468.s001.docx]

**Supplementary figures and captions**

**
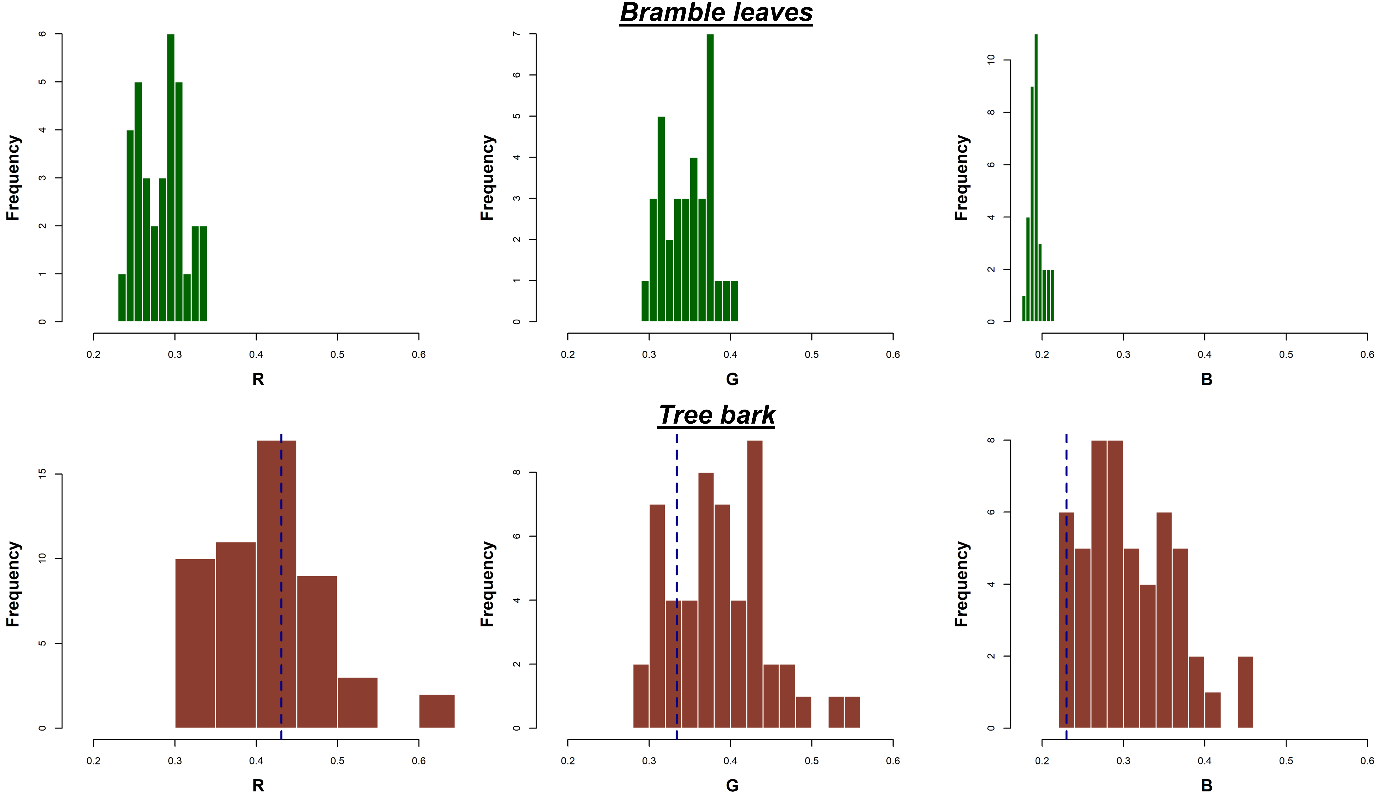
**

**Fig A.** Distributions of bird vision-calibrated normalised RGB reflectance values obtained from raw multispectral images of bramble leaves (top) and tree bark (bottom), which informed the colour of the targets used in the field predation experiment. Vertical dashed blue lines in the bottom row indicate the mean RGB values of a random sample of recently thawed mealworms (*Tenebrio molitor* larvae), which were used as bait in the experiment and fall within the RGB distribution of the tree bark - the substrate upon which the targets were pinned. All data underlying this figure can be found in https://doi.org/10.5281/zenodo.14585161

**
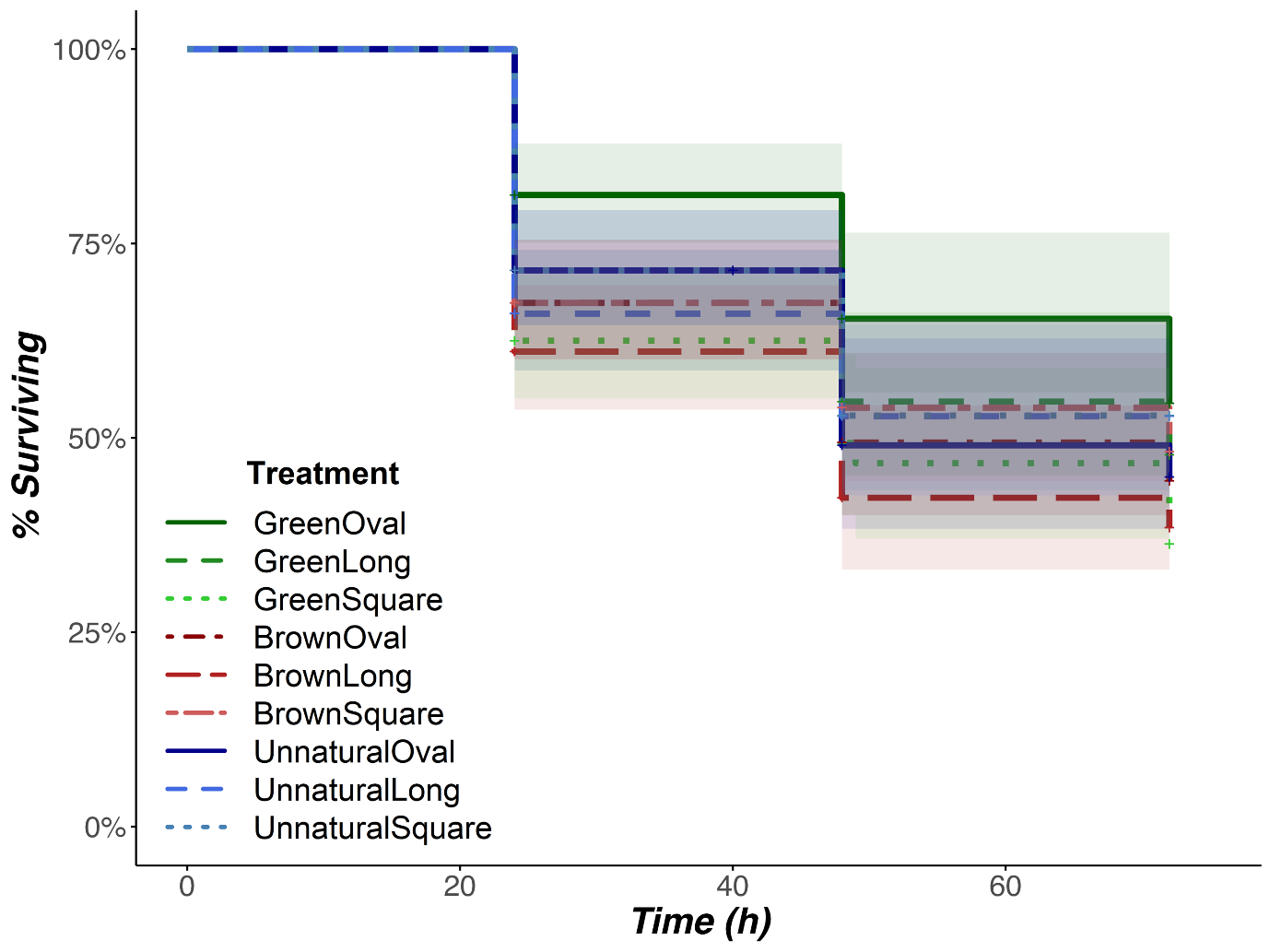
Fig B.** Survival plot for each colour x shape treatment combination over time when exposed to wild avian predation (*N* = 1,296). Shading represents 95% confidence intervals for each treatment. All data underlying this figure can be found in https://doi.org/10.5281/zenodo.14585161


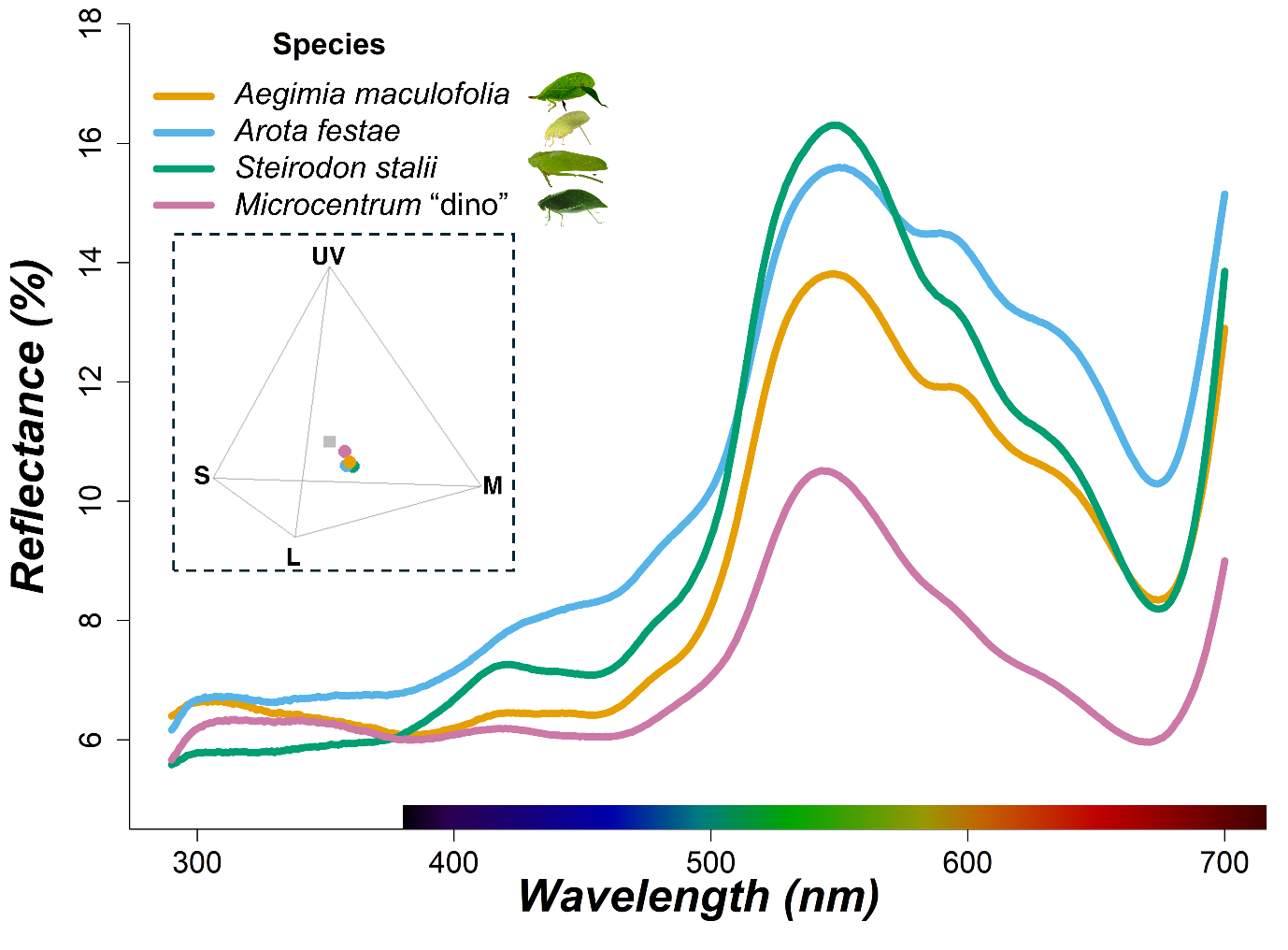


**Fig C.** Mean reflectance values for the tegmina of four randomly katydid species within a 290 to 700 nm spectral range (n = 2 per species). The inset plots these data in tetrachromatic avian colour space using the blue tit (*Cyanistes caeruleus*) visual model, where points represent the photon catch of the blue tit shortwave (S), mediumwave (M), longwave (L) and ultraviolet (UV) cones. All data underlying this figure can be found in https://doi.org/10.5281/zenodo.14585161


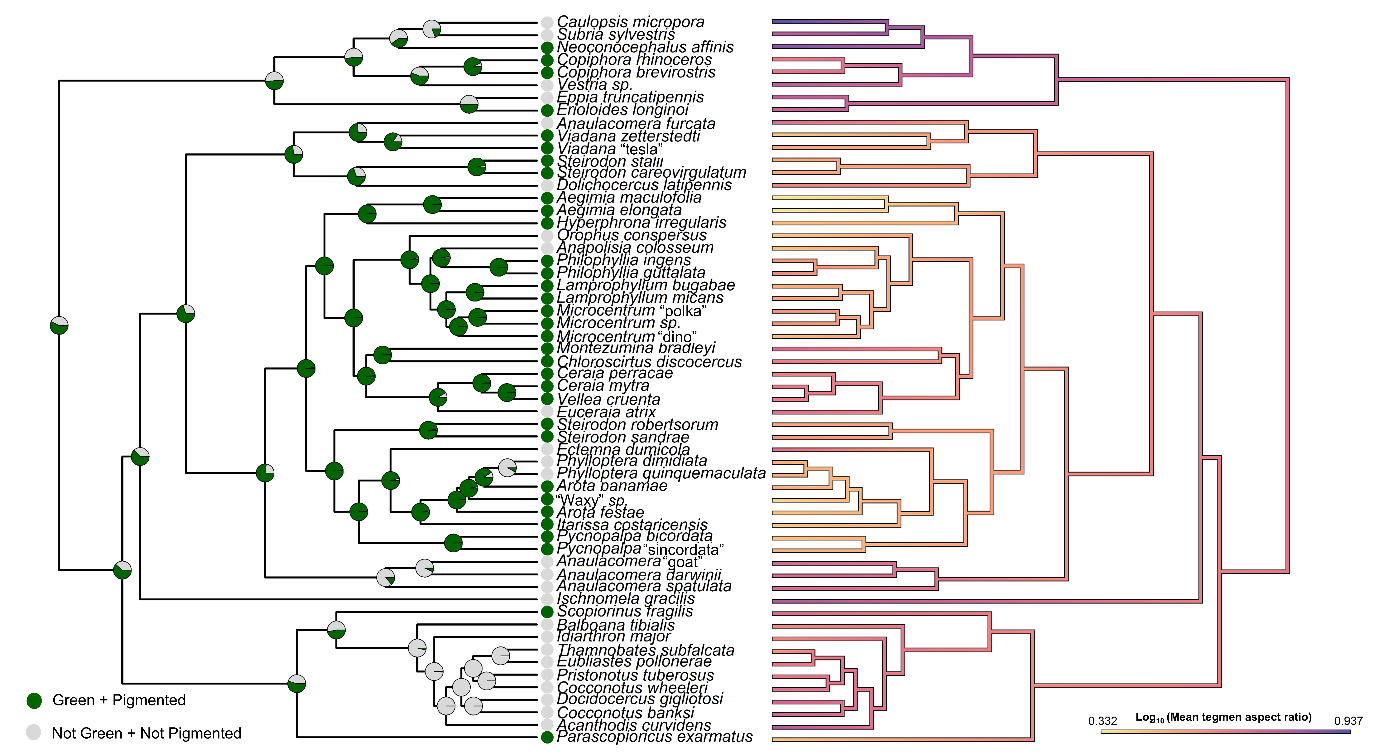


**Fig D.** Estimated transitions from absence (grey nodes and tips) to presence (green nodes and tips) of green tegmen pigmentation, and *vice versa* (left), and patterns of mean tegmen aspect ratio (au) evolution (right) across a pruned molecular phylogeny of 58 sampled katydid species [24]. Pies at each node represent the posterior probability of the ancestor having each character state. All data underlying this figure can be found in https://doi.org/10.5281/zenodo.14585161
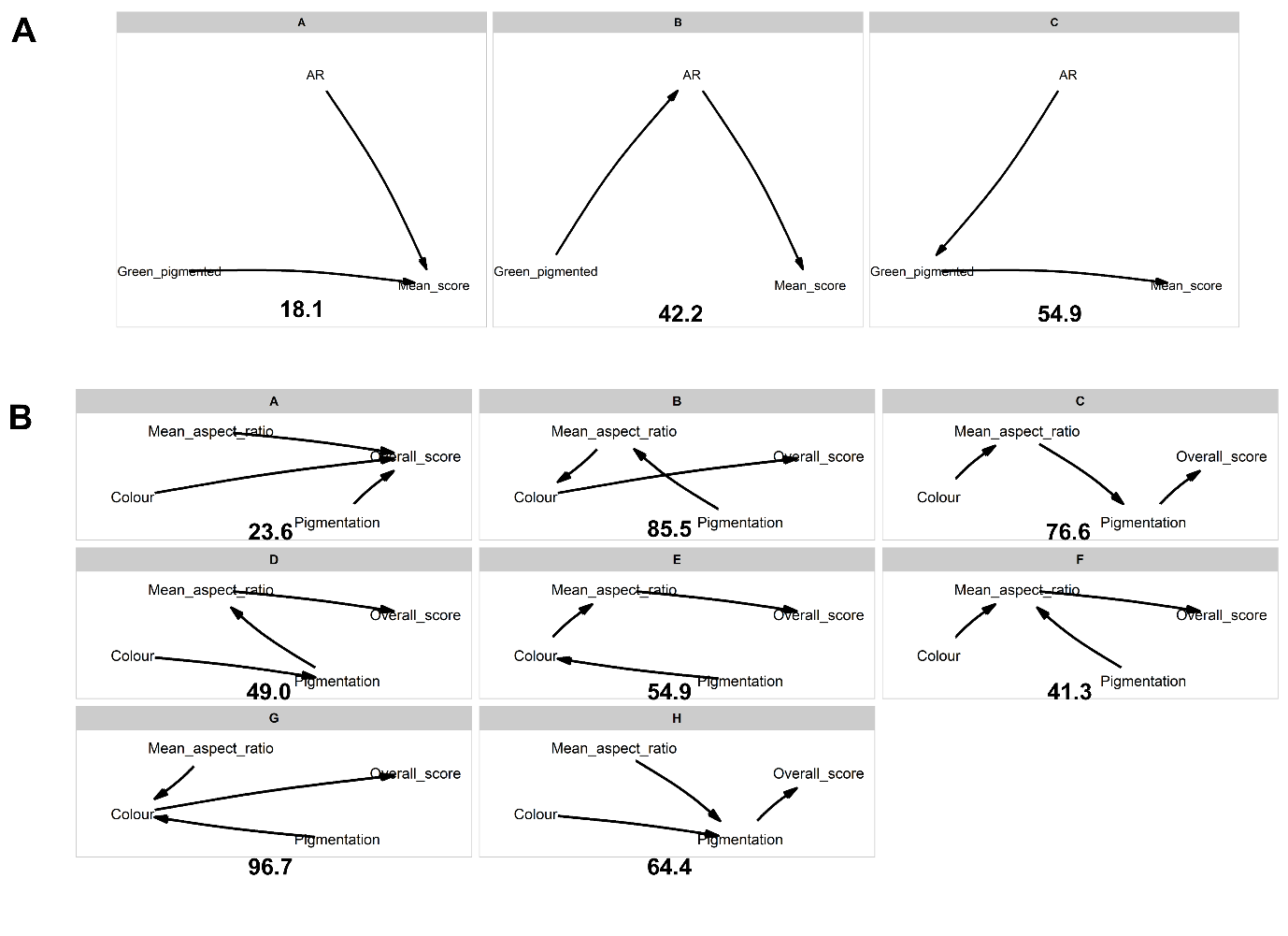


**Fig E.** All models included in each set of the phylogenetic path analysis with mean leafiness score as the response variable. The C-statistics information criterion (CIC) is indicated below each model. (A) Model set included the presence or absence of green pigmentation (Green_pigmented) and mean tegmen aspect ratio (AR) as predictor variables. (B) Model set included mean tegmen aspect ratio and the presence or absence of pigmentation, and green colouration (‘Colour’) as individual predictor variables. All data underlying this figure can be found in https://doi.org/10.5281/zenodo.14585161

**Supplementary tables and captions**

**Table A.** Results from post-hoc custom pairwise comparisons testing the effect of colour on the survival of oval shaped targets in the wild avian predation experiment, the only shape treatment where colour had a significant effect. The survival rate of the ‘green’ treatment was compared with that of the ‘brown’ and ‘blue’ colour treatments. Significant differences are denoted by asterisks. *p < 0.05, **p < 0.01, ***p < 0.001.

| **Pairwise treatment contrast** | **Estimated coefficient** | **Standard error** | **z value** | **p value** |
| --- | --- | --- | --- | --- |
| ‘Green oval’ – ‘Brown oval’ | -0.498 | 0.203 | -2.456 | 0.014* |
| ‘Green oval’ – ‘Blue oval’ | -0.438 | 0.209 | -2.090 | 0.037* |

**Table B.** Summary data for all 58 sampled species on Barro Colorado Island, Panama (*N =288*).

| **Species** | **Subfamily** | **Sample size** | | **Colouration** | **Mean tegmen aspect ratio** | **Leaf masquerade probability** | **Mean leafiness score** |
| --- | --- | --- | --- | --- | --- | --- | --- |
|  |  | ***Male*** | ***Female*** |  |  |  |  |
| *Acanthodis curvidens* | Pseudophyllinae | 1 | 0 | Brown+Pigmented | 6.417 | 0.241 | 2.574 |
| *Aegimia elongata* | Phaneropterinae | 2 | 0 | Green+Pigmented | 2.152 | 1.000 | 8.400 |
| *Aegimia maculofolia* | Phaneropterinae | 7 | 0 | Green+Pigmented | 2.147 | 1.000 | 9.074 |
| *Anapolisia colossea* | Phaneropterinae | 3 | 0 | Green+Not Pigmented | 2.737 | 0.333 | 3.130 |
| *Anaulacomera darwinii* | Phaneropterinae | 0 | 4 | Green+Not Pigmented | 5.099 | 0.547 | 4.185 |
| *Anaulacomera furcata* | Phaneropterinae | 5 | 0 | Green+Not Pigmented | 5.036 | 0.527 | 4.491 |
| *Anaulacomera* “goat” | Phaneropterinae | 2 | 2 | Green+Not Pigmented | 5.231 | 0.259 | 2.963 |
| *Anaulacomera spatulata* | Phaneropterinae | 3 | 6 | Green+Not Pigmented | 5.134 | 0.491 | 3.727 |
| *Arota festae* | Phaneropterinae | 4 | 1 | Green+Pigmented | 2.751 | 0.964 | 7.236 |
| *Arota panamae* | Phaneropterinae | 5 | 0 | Green+Pigmented | 3.147 | 0.873 | 5.600 |
| *Balboana tibialis* | Pseudophyllinae | 1 | 1 | Brown+Pigmented | 3.938 | 0.926 | 6.564 |
| *Caulopsis micropora* | Conocephalinae | 0 | 1 | Green+Not Pigmented | 8.642 | 0.057 | 1.868 |
| *Ceraia mytra* | Phaneropterinae | 1 | 1 | Green+Pigmented | 4.551 | 0.722 | 4.909 |
| *Ceraia perracae* | Phaneropterinae | 2 | 4 | Green+Pigmented | 4.344 | 0.909 | 6.800 |
| *Chloroscirtus discocercus* | Phaneropterinae | 4 | 3 | Green+Pigmented | 4.444 | 0.774 | 5.296 |
| *Cocconotus banksi* | Pseudophyllinae | 0 | 1 | Brown+Not Pigmented | 5.309 | 0.363 | 3.491 |
| *Cocconotus wheeleri* | Pseudophyllinae | 6 | 2 | Brown+Pigmented | 4.938 | 0.600 | 4.145 |
| *Copiphora brevirostris* | Conocephalinae | 5 | 1 | Green+Pigmented | 4.547 | 0.563 | 4.600 |
| *Copiphora rhinoceros* | Conocephalinae | 0 | 1 | Green+Pigmented | 4.350 | 0.660 | 5.037 |
| *Docidocercus gigliotosi* | Pseudophyllinae | 7 | 1 | Brown+Pigmented | 5.315 | 0.370 | 3.574 |
| *Dolichocercus latipennis* | Phaneropterinae | 3 | 0 | Brown+Pigmented | 4.053 | 0.722 | 4.727 |
| *Ectemna dumicola* | Phaneropterinae | 1 | 2 | Green+Not Pigmented | 4.788 | 0.593 | 4.796 |
| *Eppia truncatipennis* | Conocephalinae | 0 | 1 | Brown+Pigmented | 5.996 | 0.333 | 3.145 |
| *Erioloides longinoi* | Conocephalinae | 0 | 4 | Green+Pigmented | 5.540 | 0.636 | 4.907 |
| *Eubliastes pollonerae* | Pseudophyllinae | 0 | 1 | Brown+Pigmented | 4.752 | 0.418 | 4.036 |
| *Euceraia atryx* | Phaneropterinae | 3 | 4 | Green+Not Pigmented | 4.839 | 0.618 | 4.593 |
| *Hyperphrona irregularis* | Phaneropterinae | 1 | 0 | Green+Pigmented | 2.872 | 0.907 | 6.727 |
| *Idiarthron major* | Pseudophyllinae | 2 | 1 | Brown+Pigmented | 4.019 | 0.745 | 5.436 |
| *Ischnomela gracilis* | Pseudophyllinae | 2 | 0 | Brown+Pigmented | 6.315 | 0.291 | 3.291 |
| *Itarissa costaricensis* | Phaneropterinae | 8 | 0 | Green+Pigmented | 2.712 | 0.963 | 7.163 |
| *Lamprophyllum bugabae* | Phaneropterinae | 3 | 0 | Green+Pigmented | 3.059 | 0.907 | 6.382 |
| *Lamprophyllum micans* | Phaneropterinae | 10 | 0 | Green+Pigmented | 3.365 | 0.962 | 7.463 |
| *Microcentrum* “dino” | Phaneropterinae | 11 | 0 | Green+Pigmented | 2.819 | 0.927 | 7.109 |
| *Microcentrum* “polka” | Phaneropterinae | 16 | 7 | Green+Pigmented | 3.610 | 0.909 | 6.600 |
| *Microcentrum sp.* | Phaneropterinae | 7 | 6 | Green+Pigmented | 3.708 | 0.981 | 6.945 |
| *Montezumina bradleyi* | Phaneropterinae | 0 | 3 | Green+Not Pigmented | 5.117 | 0.667 | 4.582 |
| *Neoconocephalus affinis* | Conocephalinae | 3 | 6 | Green+Pigmented | 7.688 | 0.291 | 3.218 |
| *Orophus conspersus* | Phaneropterinae | 1 | 2 | Brown+Pigmented | 2.694 | 0.782 | 5.500 |
| *Parascopioricus exarmatus* | Pseudophyllinae | 1 | 0 | Green+Pigmented | 2.642 | 0.900 | 7.273 |
| *Philophyllia guttalata* | Phaneropterinae | 1 | 0 | Green+Pigmented | 4.074 | 0.600 | 5.545 |
| *Philophyllia ingens* | Phaneropterinae | 8 | 0 | Green+Pigmented | 3.540 | 1.00 | 7.745 |
| *Phylloptera dimidiata* | Phaneropterinae | 5 | 3 | Green+Not Pigmented | 3.096 | 0.782 | 5.345 |
| *Phylloptera quinquemaculata* | Phaneropterinae | 3 | 2 | Green+Not Pigmented | 3.062 | 0.600 | 5.455 |
| *Pristonotus tuberosus* | Pseudophyllinae | 1 | 1 | Brown+Pigmented | 4.517 | 0.352 | 3.204 |
| *Pycnopalpa bicordata* | Phaneropterinae | 2 | 0 | Green+Pigmented | 2.719 | 0.982 | 8.527 |
| *Pycnopalpa* “sincordata” | Phaneropterinae | 3 | 1 | Green+Pigmented | 2.982 | 1.000 | 7.091 |
| *Scopiorinus fragilis* | Pseudophyllinae | 1 | 0 | Green+Pigmented | 4.883 | 0.900 | 6.700 |
| *Steirodon careovirgulatum* | Phaneropterinae | 4 | 0 | Green+Pigmented | 3.197 | 0.981 | 7.527 |
| *Steirodon robertsorum* | Phaneropterinae | 3 | 0 | Green+Pigmented | 3.219 | 0.981 | 7.981 |
| *Steirodon sandrae* | Phaneropterinae | 1 | 0 | Green+Pigmented | 3.548 | 0.982 | 7.491 |
| *Steirodon stalii* | Phaneropterinae | 9 | 2 | Green+Pigmented | 3.126 | 1.000 | 8.309 |
| *Subria sylvestris* | Conocephalinae | 1 | 4 | Green+Not Pigmented | 6.041 | 0.000 | 2.764 |
| *Thamnobates subfalcata* | Pseudophyllinae | 3 | 1 | Brown+Pigmented | 3.685 | 0.455 | 3.636 |
| *Vellea cruenta* | Phaneropterinae | 1 | 0 | Green+Pigmented | 4.337 | 0.585 | 4.444 |
| *Vestria sp.* | Conocephalinae | 2 | 0 | Green+Not Pigmented | 6.192 | 0.185 | 2.109 |
| *Viadana* “tesla” | Phaneropterinae | 1 | 0 | Green+Pigmented | 3.206 | 0.660 | 4.759 |
| *Viadana zetterstedti* | Phaneropterinae | 8 | 6 | Green+Pigmented | 2.564 | 0.873 | 5.982 |
| “Waxy” *sp.* | Phaneropterinae | 9 | 6 | Green+Pigmented | 2.441 | 0.925 | 6.759 |

**Table C.** Transition rates between colouration character states across the katydid phylogeny. Included are the log likelihood and AIC values of the equal rates (null) model and the all-rates-different model. Output from log likelihood ratio tests, comparing the two models are also shown where a significant difference (p < 0.05) confirms that the all-rates-different model is accepted.

| **Trait** | **Character state** | **Transition rate to other state** | **Model log likelihood / AIC** | | **Log likelihood ratio** | **p value** |
| --- | --- | --- | --- | --- | --- | --- |
|  |  |  | **Equal** | **All-rates-different** |  |  |
| Green pigmentation  (ƛ = 1.000) | Green + Pigmented | 3.292 | -33.040 /  68.079 | -32.566  / 69.133 | 0.946 | 0.331 |
|  | Not Green + Not Pigmented | -3.292 |  |  |  |  |
| Pigmentation  (ƛ = 0.728) | Pigmented | 1.567 | -27.691 /  57.382 | -27.608  /61.217 | 0.166 | 0.684 |
|  | Not Pigmented | -1.567 |  |  |  |  |

**Table D.** Evolutionary modelling of continuous traits across the katydid phylogeny. Included are log likelihood and AIC values for Brownian motion (BM), Ornstein-Uhlenbeck (OU), and early burst (EB) models. Output from likelihood ratio tests, comparing each model with the one with the lowest AIC score are also shown.

| **Trait** | **Model log likelihood / AIC** | | | **Log likelihood ratio / p value** | | |
| --- | --- | --- | --- | --- | --- | --- |
|  | **BM** | **OU** | **EB** | **BM vs. OU** | **BM vs. EB** | **OU vs. EB** |
| Mean tegmen aspect ratio  (λ = 1.042) | 55.225 /  -106.232 | 55.225 /  -104.006 | 55.445 /  -104.446 | 0.000 / 1.000 | 0.440 / 0.507 | 0.440 / 0.507 |
| Leaf masquerade probability  (λ = 0.712) | -0.897 /  6.012 | 1.377 /  3.690 | -0.897 /  8.238 | 4.548 / 0.033 | 0.000 / 1.000 | 4.548 / 0.033 |
| Mean leafiness score  (λ = 0.655) | -119.082 /  242.383 | -116.266 /  238.976 | -119.082 /  244.609 | 5.633 / 0.018 | 0.000 / 1.000 | 5.633 / 0.018 |

**Table E.** Results from MCMCglmms which test the effect of colouration (green pigmented, pigmented), shape (mean tegmen aspect ratio) and their interaction on individual human leafiness perception (Leaf masquerade presence and Leafiness score). Shown are the posterior means, 95% credible intervals, and the effective sample size for each independent variable in the model. Significant effects are denoted by asterisks. *P_MCMC_ < 0.05, ** P_MCMC_ < 0.01, *** P_MCMC_ < 0.001.

| **Dependent variable** | **Independent variable** | **Posterior mean** | **Lower 95% CI** | **Upper 95% CI** | **Effective sample size** | **P_MCMC_ value** |
| --- | --- | --- | --- | --- | --- | --- |
| Leaf masquerade presence | Green pigmentation | 3.267 | 0.304 | 6.490 | 10000.000 | 0.037* |
|  | Mean tegmen aspect ratio | -9.455 | -13.511 | -5.464 | 10000.000 | <0.001*** |
|  | Green pigmentation * Mean tegmen aspect ratio | -2.109 | -7.090 | 2.954 | 10000.000 | 0.406 |
| Leafiness score | Green pigmentation | 1.020 | -0.306 | 2.396 | 9596  .000 | 0.142* |
|  | Mean tegmen aspect ratio | -8.985 | -11.063 | -6.992 | 9504  .000 | <0.001*** |
|  | Green pigmentation * Mean tegmen aspect ratio | 0.349 | -2.100 | 2.589 | 9690  .000 | 0.768 |
| Leaf masquerade presence | Pigmentation | 4.7364 | 1.005 | 8.674 | 10000.000 | 0.017* |
|  | Mean tegmen aspect ratio | -6.790 | -12.611 | -0.630 | 10310  .000 | 0.026* |
|  | Pigmentation * Mean tegmen aspect ratio | -4.772 | -10.805 | 1.531 | 10000.000 | 0.120 |
| Leafiness score | Pigmentation | 3.362 | 1.069 | 5.588 | 9650.000 | 0.005* |
|  | Mean tegmen aspect ratio | -4.986 | -8.799 | -1.278 | 9680.000 | 0.010* |
|  | Pigmentation * Mean tegmen aspect ratio | -3.840 | -7.522 | -0.136 | 9632.000 | 0.045* |
| Leafiness score | Mean aspect ratio (Pigmented species) | -8.576 | -12.175 | -5.317 | 11234.000 | <0.001*** |
|  | Mean aspect ratio (Non pigmented species) | -6.144 | -11.705 | -9.959 | 10000.000 | 0.025* |

**Table F.** Results from phylogenetic generalized least-squares (PGLS) models which test the effect of colouration (green pigmented, pigmented), shape (mean tegmen aspect ratio) and their interaction on the species means of two metrics of human leafiness perception (Leaf masquerade probability and Mean leafiness score). Phylogenetic signal of each model was calculated using the maximum likelihood estimate of Pagel’s λ. Significant effects are denoted by asterisks. *p < 0.05, **p < 0.01, ***p < 0.001.

| **Dependent variable** | **Independent variable** | **Pagel’s λ** | **Estimated coefficient** | **Standard error** | **t value** | **p value** |
| --- | --- | --- | --- | --- | --- | --- |
| Leaf masquerade probability | Green pigmentation | 0.000 | 0.141 | 0.185 | 0.762 | 0.450 |
|  | Mean tegmen aspect ratio |  | -1.234 | 0.223 | -5.540 | <0.001*** |
|  | Green pigmentation * Mean tegmen aspect ratio |  | 0.160 | 0.294 | 0.546 | 0.588 |
| Leafiness score | Green pigmentation | 0.000 | 2.792 | 1.278 | 2.185 | 0.033* |
|  | Mean tegmen aspect ratio |  | -6.575 | 1.535 | -4.283 | <0.001*** |
|  | Green pigmentation * Mean tegmen aspect ratio |  | -1.931 | 2.025 | -0.954 | 0.344 |
| Leaf masquerade probability | Pigmentation | 0.000 | 0.512 | 0.152 | 3.359 | 0.001** |
|  | Mean tegmen aspect ratio |  | -0.077 | 0.028 | -2.752 | 0.008** |
|  | Pigmentation * Mean tegmen aspect ratio |  | -0.065 | 0.031 | -2.083 | 0.042* |
| Leafiness score | Pigmentation | 0.000 | 3.790 | 1.133 | 3.346 | 0.001** |
|  | Mean tegmen aspect ratio |  | -0.481 | 0.204 | -2.362 | 0.022* |
|  | Pigmentation * Mean tegmen aspect ratio |  | -0.536 | 0.230 | -2.334 | 0.023* |
| Leafiness score | Mean tegmen aspect ratio (Pigmented species) | 0.564 | -0.961 | 0.159 | -6.053 | <0.001*** |
|  | Mean tegmen aspect ratio (Non pigmented species) | 0.000 | -0.531 | 0.161 | -3.298 | 0.008** |

**Table G.** Path coefficients from the best fitting model from four separate phylogenetic path analysis model sets, alongside the standard error and 95% confidence intervals.

| **Model set** | **Path** | **Path coefficient** | **Standard error** | **Lower 95% confidence interval** | **Upper 95% confidence interval** |
| --- | --- | --- | --- | --- | --- |
| Green pigmentation (GP), mean tegmen aspect ratio (AR), leaf masquerade probability (LP) | GP -> LP | 0.877 | 0.137 | 0.633 | 1.140 |
|  | AR -> LP | -0.602 | 0.068 | -0.760 | -0.470 |
| Green pigmentation (GP), mean tegmen aspect ratio (AR), leafiness score (LS) | GP -> LS | 0.892 | 0.149 | 0.612 | 1.172 |
|  | AR -> LS | -0.571 | 0.075 | -0.716 | -0.431 |
| Colour (C), Pigmentation (P), mean tegmen aspect ratio (AR), leaf masquerade probability (LP) | C -> LP | 0.895 | 0.156 | 0.429 | 1.020 |
|  | P -> LP | 0.728 | 0.166 | 0.579 | 1.221 |
|  | AR -> LP | -0.609 | 0.069 | -0.760 | -0.489 |
| Colour (C), pigmentation (P), mean tegmen aspect ratio (AR), leafiness score (LS) | C -> LS | 0.756 | 0.173 | 0.421 | 1.060 |
|  | P -> LS | 0.861 | 0.183 | 0.503 | 1.224 |
|  | AR -> LS | -0.583 | 0.076 | -0.738 | -0.442 |
