# Supplementary material for: Functional and evolutionary synergy of trait components can explain the existence of leaf masquerade in katydids
Source: PLoS Biol. 2025 Nov 3;23(11):e3003468. doi: 10.1371/journal.pbio.3003468 (PMC12582443; doi:10.1371/journal.pbio.3003468)
Supplement: Attachment — Submitted filename: Response to Reviewers.pdf [file pbio.3003468.s002.pdf]

Response to Referees

Below we provide information on how we have addressed the editor and reviewers' comments, including all editorial changes to the text.

Original reviewer comments are in black italics, the authors' responses in blue, and quotes from corrected text in blue italics. Line numbers in brackets refer to the updated manuscript, without inclusion of tracked changes.

EDITIOR COMMENTS

- *As you will see, although the reviewers think that the findings are potentially interesting and provide an advance in our understanding of the evolution of camouflage in katydids, they also note several important concerns. Specifically, Reviewer #2 raises concerns about the design of the field predation experiments and that the experiments should be repeated in Panama, as well as the conclusions regarding simultaneous and synergistic evolution as opposed to stepwise evolution. Reviewer #3 raises concerns about the design of the behavioral experiments, the overlooking of intraspecific variation and the methods used to model predator vision. Reviewer's #1 and #4 are more positive, although Reviewer #1 asks why the manuscript does not include two additional genera (Mimetica and Typophyllum) and Reviewer #4 requests additional methodological clarifications and presentational edits.*

Thank you for the opportunity to resubmit a revised version of our manuscript, and for succinctly summarising the reviewers' concerns and the priorities for revision. A lack of clarity in some parts of our original submission may have led reviewers to arrive at these concerns. To address this, we collected new data, re-ran existing analyses with the new data, ran additional new analyses, and significantly expanded on explanations and justifications for our technical and analytical approaches. With these edits, our key conclusions supporting synergistic functionality and evolution of leaf masquerade in katydids are strengthened. We thank the reviewers and editor for their thoughtful critique which helped us improve the robustness and general interest of our results.

Specifically, we have:

- Included data on an additional seven katydid species (sampled since the original submission of our manuscript), some of which were specifically suggested by reviewer 1. Incorporating these new individuals involved updating the human online survey and rerunning all phylogenetic comparative analysis. Our main conclusions are unchanged.

- Explained in detail why deliberately conducting the field predation experiment in the UK was essential for identifying the adaptive significance of leaf masquerade, and why repeating this experiment in Panama would be testing a different hypothesis, unrelated to the current manuscript.

- Substantially expanded the Results section to directly link predictions about simultaneous *vs.* stepwise evolution from the Introduction to the outputs of our statistical analyses.

- Provided detailed justification and additional analyses to support our experimental design and visual modelling approach, which is based on well-established methodological techniques that have been used in similar published studies.

- Incorporated sex into our evolutionary association analysis to better account for intraspecific variation. We have also clarified that all species used in our analyses were either monomorphic with regards to the morphological traits measured, or that intraspecific variation was already accounted for in our original analyses.

Please see our detailed responses below for further clarification. We appreciate your time and consideration, and we hope to have convinced you that the findings presented in this manuscript about concealment strategies in animals will make a significant contribution across multiple fields of biology.

REVIEWER 1

- *Here is my revision of the MS PBIOLOY-D-25-01133, entitled "Extraordinary adaptations: Functional and evolutionary synergy of trait components can explain the existence of leaf masquerade".*

  In this paper, the authors used artificial leaf-mimicking models of katydid (Orthoptera Tettigoniidae) wings, and free-living avian predators to investigate how interactions among the component traits comprising leaf masquerade affect predator perception in the wild. They use phylogenetic regression of wing morphology coded/integrated with human perception of leafiness to investigate the evolutionary origins of masquerade in the wings of some species. Results suggests that both colour and shape interaction and is necessary for leaf masquerade to be functional, and this synergistic evolution is corroborated using phylogenetic approaches.

  I enjoyed reading this paper a believe the research is a good step forward in understanding the evolution of camouflage in katydids. Most katydid species are nocturnal, which suggests that this is an adaptation to escape predation by birds during the day. The main natural enemies of this family are birds, and some primates during the day, and bats and rodents at night. It is clear that the diversity in wing morphology and camouflage observed across species is an adaptation to evade visual predators. This paper focuses on the three major subfamilies of Tettogoniidae, Phaneropterinae, Pseudophyllinae, and Conocephalinae, using 51 neotropical species from Panama. While these species constitute a good representation of wing diversity, my main comment goes to why the authors didn't include the genus Mimetica and Typophyllum? These genera are perhaps the most dramatic examples of masquerade, with bizarre wings that simulate necrotic leaves, including shapes that imitate leaves bitten by insects. Species within this genera include green and brown forms, and in this case I wonder how would you have coded these in terms of coloration transition and tegmen ratio? Would this affect the evolutionary association between tegmen aspect ratio shown in Fig. 2? For what I can see here in our collection the wing aspect ratio of these insects is below 2.

We thank the reviewer for their thoughtful comments and accurate interpretation of our work. Species within the genera *Mimetica* and *Typophyllum* are indeed impressive leaf-masqueraders. Although *Mimetica* had been previously recorded at our field site on Barro Colorado Island (BCI), Panama, we did not encounter any individuals during our 2024 collecting trip. *Typophyllum* were not sampled as, to our knowledge, the distribution of this genus is restricted to South America at higher altitude forest than BCI so is not locally present.

In addition, you are correct that these genera, unlike all other species in our comparative analysis, are extremely polymorphic for both colour and shape, to the extent that no two individuals are alike. To our knowledge, it is not known whether these differences are

heritable or the result of phenotypic plasticity. As such, assigning species-level categorical colour states or summarising shape variation accurately is currently unfeasible. Robustly modelling this variation and incorporating it into our comparative analysis would require a much greater sample size.

Additionally, the phylogenetic placement of *Mimetica* and *Typophyllum* remains uncertain. Recent studies suggest that they form a distinct subfamily that is a sister group to all other katydids included in our analysis (Mugleston *et al.* 2013, *Mol Phylo Evo*; Kernan *et al.* 2024, *Integ Comp Biol*). Our initial comparative dataset thus comprises a monophyletic clade that is relatively evolutionarily distant from *Mimetica* and *Typophyllum.* In light of all the above, these genera are not included in our comparative analyses.

Nonetheless, the pronounced polymorphism observed in *Mimetica* and *Typophyllum* is intriguing. The apparent lability of colour and shape within and between species suggests evolutionary decoupling between colour and shape in this subfamily that is worthy of more targeted investigation. We agree this is worth addressing in the main text, so we have added the following to the discussion:

"The adaptive significance and evolutionary mechanisms underlying within-species polymorphisms remain an important avenue for further targeted study [40]. Colour polymorphisms in particular are common in many of our sampled species when comparing populations across their geographic range, but here we used species for which intraspecific polymorphism was absent at our sampling locality, enabling species-level phylogenetic comparative analyses. Although not part of our analysis, genera within the subfamily Pterochrozinae such as Mimetica and Typophyllum are exceptional examples of leaf masquerade, and these do exhibit striking within-species differences in both colouration and shape, even within the same population [40,41]. It is not known whether this variation is heritable or the result of environmentally induced plasticity, However, such extreme phenotypic lability might hint that colour and shape have become evolutionarily decoupled in this lineage. Testing this will require large within-species sample sizes. It would also be interesting to test if predator learning shapes the relative fitness of these morphs among communities of experienced predators." (lines 247-260)

- *In addition, in the Pseudphylliane: Pterophyllinae, there are mostly green species, with broad wings, like Lophaspis sp. Xestoptera, Scopioricus, etc. Why were these not included? They seem to be very abundant in BCI.*

While these genera are present in Panama, no individuals were encountered during our 2024 collecting trip to BCI. However, since the initial submission of our manuscript, we have sampled individuals of an additional seven species that were not sampled in 2024 and therefore not included in our original analyses. These comprise the pterophyllines *Scopiorinus fragilis* (Pseudophyllinae) and *Parascopioricus exarmatus* (Pseudophyllinae), as well as *Aegimia elongata* (Phaneropterinae), *Philophyllia guttulata* (Phaneropterinae), *Phylloptera quinquemaculata* (Phaneropterinae), *Pycnopalpa* "sincordata" (Phaneropterinae), and *Thamnobates subfalcata* (Pseudophyllinae).

To increase the robustness of our dataset and further test the stability of our original findings, we incorporated these new species into the analysis by collecting morphological data and distributing an updated online survey - including comparable lightbox images - to an additional 11 participants. After confirming that the mean scores for the overlapping species did not significantly differ between the original and new survey subsets using MCMCglmm, we pooled the data and reran all phylogenetic comparative analyses with these new species and updated leafiness scores. Survey version (i.e. whether or not it included the additional species) is also now included as an additional random effect in all MCMCglmm analyses involving survey data.

The results of all analyses are essentially unchanged. The only differences are that:

- Previously, both human-based metrics of leafiness best fit a Brownian motion (BM) model of trait evolution. Now, an Ornstein-Uhlenbeck (OU) model is best fitting. This strongly indicates that, despite still showing a strong phylogenetic signal, leaf masquerade has evolved under strong directional selection across the phylogeny. In other words, our finding that leaf masquerade is under directional selection is stronger now. To convey this, we have modified the following section of the main text:

  "Modelling the human leafiness metrics in the same way showed a similarly high phylogenetic signal, but this time, an Ornstein-Uhlenbeck (OU) model was best fitting (Table S3). These results indicate a strong degree of evolutionary inertia, potentially limiting the axes of adaptive morphological evolution among leaf-masquerading species [24]. Despite these constraints, the best-fit OU model for overall leafiness suggests that this trait has evolved under strong directional selection across the phylogeny." (lines 152-157)

- Although human-assessed leafiness remains dependent on the interaction between mean aspect ratio and pigmentation, the relationship with mean aspect ratio is now significant for both pigmented and non-pigmented species in both MCMCglmm and PGLS analyses. In our previous analysis, this relationship was significant only for pigmented species. Nonetheless, the strength of the association remains markedly stronger in pigmented species. Our conclusions here therefore remain unchanged, but we updated the main text as follows:

  "When individual participant data were analysed using phylogenetic generalized linear mixed models, the interaction between mean aspect ratio and pigmentation predicted the human leafiness score, echoing results from the avian predation experiment (MCMCglmm: P-mean = -3.840, 95% CI = -7.522 – -0.136, P_{MCMC} = 0.045; Figure 2A,C; Table S4). When species were subset by pigmentation status, participants consistently awarded high leafiness scores to low aspect ratio tegmina, but the strength of this association was stronger for pigmented species (MCMCglmm: pigmented, P-mean = -8.930, 95% CI = -10.495 – -7.234, P_{MCMC} < 0.001; not pigmented, P-mean = -6.144, 95% CI = -11.705 – -9.959, P_{MCMC} = 0.025)." (lines 171-178)

We updated the main text, figures and relevant supplementary tables accordingly to reflect the inclusion of these new species and include the following new text in the Methods:

"Adult katydids were sampled at night from lights around research station buildings on Barro Colorado Island (BCI), Panama (9.1647° N, 79.8367° W) in March-April 2024 and January-May 2025 between 04:00 and 06:00 and 20:00 and 01:00. Individuals were identified to species level using public resources [44,45] and custom ID keys provided by Dr Laurel Symes and Dr Hannah ter Hofstede. A total of 288 individuals were sampled across 58 species (51 species were sampled in 2024, with a further 7 species sampled in 2025), with representatives…" (lines 301-306)

"In June 2025, an updated version of the survey incorporating an additional seven species sampled in 2025 was completed by a further 11 participants (55% female, aged 18+, all with trichromatic colour vision)." (lines 480-482)

"Species, participant, and survey version were included as additional random effects and each model was run for 5,100,00 iterations, with a burnin of 100,000. A separate model also

confirmed that participant responses did not significantly differ between the two versions of the survey conducted in 2024 and 2025 (leaf masquerade presence: P-mean = -0.699, 95% CI = -1.899 – 0.416, P_{MCMC} = 0.238; leafiness score: P-mean = -0.182, 95% CI = -0.950 – 0.561, P_{MCMC} = 0.651)." (lines 522-527)

- *General comments*

  Lines 28-30. Abstract. Make this last sentence most digestible for the lay reader.

We have rephrased this sentence to make it more accessible. It now reads as follows:

"Our findings identify the adaptive value of masquerade camouflage in the wild and that concordant evolutionary change in separate traits – evolutionary synergy - can generate extraordinarily specialised, multi-component adaptations such as leaf masquerade." (lines 28-31)

- *Line 204-206. I think you are missing citing a vital paper where all of these morphological patterns of wing masquerade are discussed for Typophyllum. Baker A., Sarria-S FA, Jonsson T., Morris G.K. & Montealegre-Z F. 2017. Wing resonances in a new dead-leaf mimic katydid (Tettigoniidae: Pterochrozinae) from the Andean cloud forests. Zoologischer Anzeiger- Journal of Comparative Zoology. 270: 60-70*

Thanks for the suggestion. This reference has now been added (line 245).

- *Line 341. Wouldn't the brown colour of the mealworm contrasts with the green shapes?*

We thank the reviewer for raising this point. Because mealworms are brown in colouration, we assumed that they would have matched the brown bark background upon which targets were pinned, and therefore, not interfere with how treatment groups were perceived. We have now confirmed this by taking bird-calibrated photographic images of a random sample of recently thawed mealworms (from the same supplier as those used in our original experiment). By applying the same visual modelling and calibration techniques used to create the original targets, we confirm that the mean RGB reflectance values for these mealworms falls within the RGB distribution for the tree bark we measured. We also note that several published studies have conducted similar predation experiments where targets are pinned with the bait (usually edible mealworms) completely exposed (e.g. see Costello *et al.* 2020, *Proc R Soc B;* Cuthill *et al.* 2005, *Nature*; Stevens *et al.* 2007, *Anim Behav;* Yeager *et al.* 2024, *Ecol & Evol*). To illustrate this additional analysis, we now plot the distribution of these calibrated RGB values in the following supplementary figure (Figure S1):

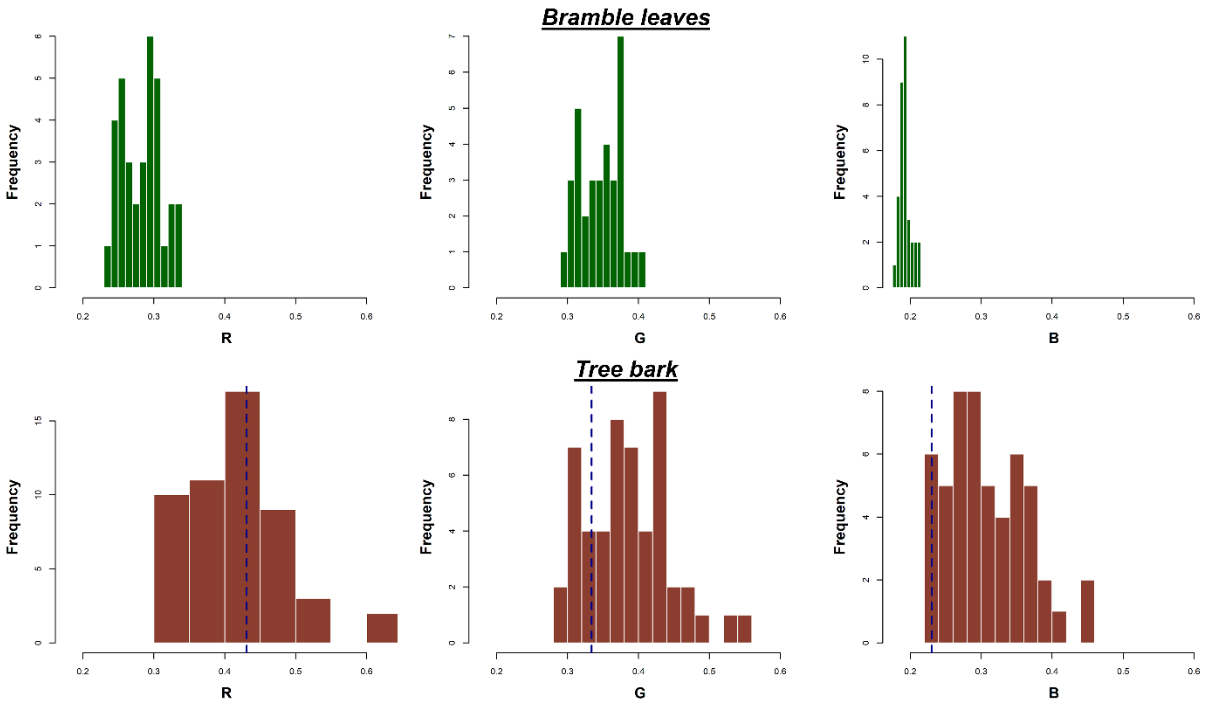

*“**Figure S1.** Distributions of bird vision-calibrated normalised RGB reflectance values obtained from raw multispectral images of bramble leaves (top) and tree bark (bottom), which informed the colour of the targets used in the field predation experiment. Vertical dashed blue lines in the bottom row indicate the mean RGB values of a random sample of recently thawed mealworms (*Tenebrio molitor *larvae), which were used as bait in the experiment and fall within the RGB distribution of the tree bark - the substrate upon which the targets were pinned.”* (lines 760-767)

We have also now added the following to the Methods:

“To confirm that the addition of the brown mealworm bait would not meaningfully affect the relative contrast of the different treatments against their background, a set of recently thawed mealworms was photographed under the same lighting conditions and calibrated in the same way. As expected, the mean reflectance values for the mealworms fell within the RGB distribution of the tree bark background upon which targets were pinned (Figure S1).” (lines 363-368)

- *Figures:*
  Figure 2. Panel A. The picture of the inset depicting a Pseudophyllinae katydid is not a Pseudophyllinae. It is actually Eppia truncatipennis, a Conocephaloid (Agraeciini). Please replace this inset with a picture of a real Pseudophyllinae.

Thanks for bringing this to our attention. The image of *Eppia truncatipennis* has now been swapped for one of *Cocconotus wheeleri* (Pseudophyllinae). The figure now looks as follows:

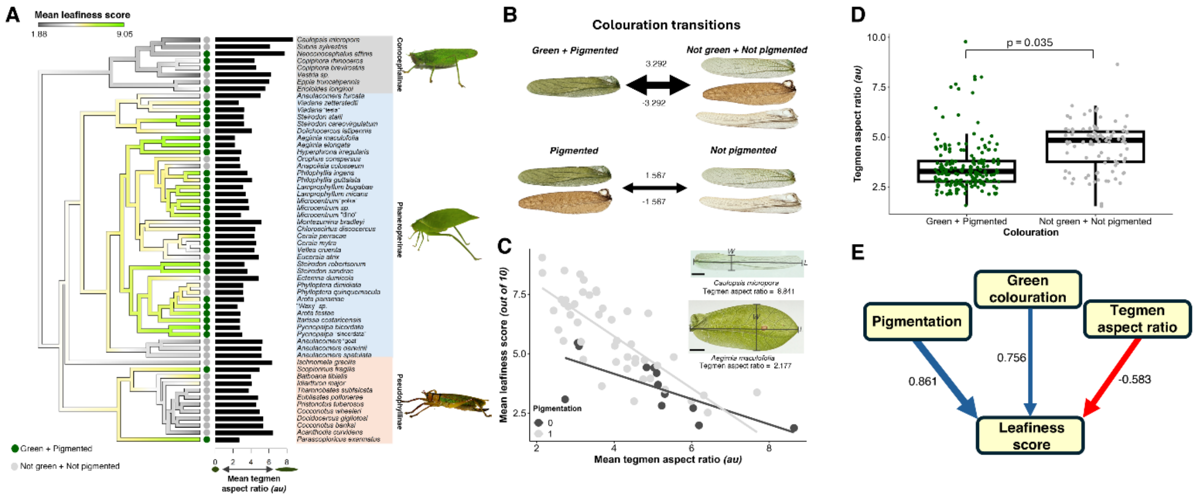

REVIEWER 2

- *The manuscript by Wainwright and Colleagues reports on the fascinating biology of leaf masquerade in Katydids. Specifically, they claim to test between two alternative evolutionary hypotheses for the origin of complex adaptations: whether the component traits originated via non-adaptive, intermediate evolutionary steps or via simultaneous, synergistic evolution. Using field predation experiment, human scoring of "leafiness", and phylogenetic comparative analyses across 51 Katydid species, the Authors argue that simultaneous and synergistic evolution between individual traits "fosters evolution of extraordinarily specialised adaptations." As a reader, I had a difficult time assessing the novelty and robustness of the conclusions, largely because of the short format of the manuscript (many of the important details are in the Methods) and the grandiose writing in some places. In general, the Authors did a poor job of connecting each individual question, to the alternative possible hypotheses, to the method, and then to the result for the Reader. Here are my major concerns that I feel need to be addressed before this manuscript can be considered for publication:*

  Given the existing work on Katydids (Mugleston et al. 2016), which reconstructs ancestral states of leaf masquerade evolution, and butterflies (Suzuki et al. 2014), which addresses the question of whether component traits are stepwise or simultaneous, the novelty or advance of the current work hangs on three points: (1) they conclude that it simultaneous and synergistic evolution whereas previous studies claim stepwise evolution; (2) they integrated the use field predation experiments; and (3) they used human scoring of leafiness as proxies for birds recognition systems. I will therefore evaluate the strength of the evidence for each of these points:

We thank this reviewer for the clear synthesis of their main concerns, which has helped us to identify areas lacking clarity in our original submission. We address all of these concerns in detail below.

- *Concerns about conclusion of simultaneous and synergistic evolution:*

  >> The set up of the phylogenetic path analysis is not entirely clear. This reader was expecting clear predictions about which paths would be considered step-wise and which paths would be considered simultaneous and synergistic. This should be made more clear both in the Methods and in the Main text.

We agree that we were overly brief in our original description of the phylogenetic path analysis. We now elaborate on the method in the Main Text starting at line 211. To summarise, phylogenetic path analysis (PPA) disentangles the most likely sequence by which traits are acquired across a phylogeny by constructing different evolutionary scenarios using structural equation modelling. In the case of the leaf masquerade traits we studied, the models constructed within any particular PPA included scenarios where the initial acquisition of trait components (i.e. colouration and shape) preceded one another in a stepwise fashion across the phylogeny, as well as a scenario where both traits were acquired concurrently. For all PPA we performed, the latter model had consistently the best fitting causal structure (based on CIC score). Hence, simultaneous, synergistic acquisition of colouration and shape best explains the evolution of leaf masquerade in our studied species.

We have now significantly elaborated on our description of this in the main text as follows:

"Given the evolutionary correlation between trait components, we next assessed the order of pigmentation, green colouration, and aspect ratio acquisition across the phylogeny using phylogenetic path analysis. This method involved constructing structural equation models (SEMs) to represent alternative evolutionary sequences by which these traits were acquired stepwise across the phylogeny. We also modelled a scenario in which all three traits were acquired concurrently, as well as hybrid models that combined elements of simultaneous and stepwise acquisition of the three traits. When either human-perceived metrics of leafiness were used as the dependent variable, the path analysis yielded strong support for models of simultaneous acquisition of all three traits, supporting their coordinated, synergistic evolution during the origin of leaf masquerade (Figure 2E; Figure S5; Table S6). Taken together, the evolution of leaf masquerade in our studied species is best explained by the simultaneous, synergistic acquisition of trait components under directional selection." (lines 209-220)

We have also added the following elaboration to the methods:

"…the best fitting model structure is indicated by the model with the lowest C-statistics information criterion (CIC). A best-fitting model structure whereby traits are acquired sequentially would therefore suggest stepwise evolution, whereas one where all traits are acquired concurrently would suggest simultaneous, synergistic evolution." (lines 548-551)

- *>> Furthermore, in the abstract and Introduction the alternative hypotheses are composite traits arising separately through non-adaptive versus simultaneously through directional selection. However, there is little attempt to connect the models of evolution that best fit the data to the conclusion of non-adaptive versus directional selection? What model would best fit a non-adaptive hypothesis? Brownian motion? Not sure it's so straightforward and would take clarity on this point in the manuscript.*

We agree that the predictions outlined on lines 39-50 of the introduction could have been more explicitly connected to the outcome of our evolutionary analysis in the results/discussion. Non-adaptive stepwise evolution predicts that individual trait components show no evolutionary correlation and arise independently – that is, they do not tend to arise at the same time, i.e. at the same position on the phylogeny. This is the scenario supported by the composite evolution of 'springboard trapping' in carnivorous pitcher plants (Chomicki *et al.* 2024, *Science*). By contrast, if component traits were to arise simultaneously under directional selection, we would expect them to be evolutionarily correlated – that is, arising at the same time, i.e. same position on the phylogeny. This second alternative is exactly what our MCMCglmm and phylogenetic ANOVA analyses showed. When combined with the outcome of our phylogenetic path analysis, this supports the conclusion that leaf masquerade originated via coordinated synergistic evolution of its component traits under directional selection.

We have now substantially expanded on this section to provide greater clarity on the implications of each evolutionary scenario and to more explicitly link these theoretical expectations with the outcome of our analyses. This revised section now reads:

"Given that the interaction between colouration and shape enhances the efficacy of leaf masquerade, we next tested whether non-adaptive stepwise or simultaneous acquisition of these trait components explain its evolution. Non-adaptive stepwise evolution would imply no evolutionary correlation between colouration and shape across the phylogeny – that is, these traits would not tend to arise at the same time during the evolutionary history of these species. The opposite is predicted if these traits evolved simultaneously – that is, they should tend to arise concurrently during the species' evolutionary history. After controlling for phylogeny, MCMCglmm analysis on individual data and a phylogenetic ANOVA on species means confirmed that the evolution of green pigmentation was significantly associated with reduced tegmen aspect ratios (MCMCglmm: P-mean = 0.062, 95% CI = 0.004 – 0.117, P_{MCMC} = 0.035; phylogenetic ANOVA: F_1 = 16.127, p = 0.025; Figure 2D). These results are consistent with an evolutionary scenario whereby colouration and shape simultaneously evolved under directional selection." (lines 193-205)

To address the comment regarding Brownian motion (BM), a BM model represents stochastic trait evolution where variance accumulates incrementally over time at a constant rate. Importantly, a BM process does not necessarily indicate the absence of selection, particularly across deep evolutionary timescales where even small adaptive steps in a particular direction can resemble BM when viewed at a macroevolutionary scale. Rather, a best-fitting BM model could reflect strong phylogenetic constraint on traits (e.g. functional, developmental limitations), restricting their rate of evolution. Nevertheless, although our human metrics of leafiness do show a high phylogenetic signal, our updated evolutionary modelling of these variables indicates an Ornstein-Uhlenbeck (OU) model is best fitting, which is much more strongly indicative of phylogeny-wide selection on leaf masquerade. Thus, our updated taxon sampling has resulted in stronger evidence to support our original conclusion. Please see lines 152-157 of the manuscript where we have added this information, and our response to a previous comment on page 4.

- *Concerns about field predation experiment:*

  >> I understand the rationale the Authors propose for doing the field experiment in UK, but to this reader this would be a control rather than the principal experiment. I was expecting a similar experiment to be done with predators of katydids in Panama. So, to this readers, it is important to do this experiment in places.

We appreciate this feedback and understand why a reader might question why the predation experiment was not conducted in the native habitat of leaf-mimicking katydids. There were several conceptual and practical reasons that informed our decision not to do so, which we explain in detail below. As we previously briefly outlined in the original submission (formerly lines 81-85), the main reason for conducting the predation experiment in the UK was to minimise confounding effects associated with predator learning. We now expand on this rationale at lines 84-95 of the main text, as we appreciate that other readers might have the same question.

- It is well established that birds, both in field and laboratory studies, can form search images for prey based on specific features which guide their foraging decisions (e.g. Lawrence, 1985, *Anim Behav*; Pietrewicz & Kamil, 1979, *Science*). Furthermore, evidence suggests that different camouflage strategies interfere with search image formation to varying degrees (Skelhorn & Rowe, 2016, *Proc R Soc* B; Troscianko *et al.* 2016, *Proc R Soc* B), which may affect their relative fitness depending on whether

predators are naïve or experienced. Our experiment was designed to isolate the adaptive significance of colour and shape in specifically contributing to the effectiveness of masquerade camouflage, independently from general background matching. Conducting this experiment in Panama, where the local predator community is likely to have prior experience of leaf-mimicking katydids, would therefore introduce bias through learned recognition. This would potentially enhance predation on realistic treatments (e.g. the 'green oval' treatment) if predators had learned they were profitable, while artificially lowering predation on unnatural-looking controls (i.e. the blue treatment groups) which our UK-based results already suggest elicit neophobic responses.

In summary, search image formation by experienced predators could obscure the adaptive value of the different treatment groups in a context mimicking the early evolutionary stages of masquerade, which is what our experiment was designed to test. This consideration is especially important in the context of masquerade because a key assumption underlying its definition is that prey are misclassified, rather than simply going undetected. If experienced predators have learned to classify leaf-masquerading katydids correctly (i.e. as prey), isolating the adaptive benefit of masquerade under these conditions would not be possible with our current experimental treatments. We now detail this logic at lines 84-95.

- Although comparing naïve and experienced predator communities would be interesting, such a study would address how predator experience and/or predator-prey coevolution shapes masquerade, which strays from the question we address in the current study. Additionally, our targets were specifically designed to resemble the colouration of leaves and bark of UK plant species. Repeating the same experiment in Panama – where these plants do not occur – would reduce the ecological relevance and comparability of these two experiments.

- Avian visual systems—particularly in passerines—are broadly conserved in terms of colour perception (especially longer wavelengths) and spatial resolution (e.g. Hart & Hunt, 2007, *Am Nat;* Kelber, 2019, *Curr Op Behav Sci*; Martin, 2022, *Curr Biol;* Osorio *et al.* 1999, *Evol Ecol*). As such, from a mechanistic standpoint, we have no reason to expect that our targets would be perceived any differently to the passerine bird species in the UK *versus* those present during the evolutionary history of leaf masquerade in the Neotropics. Now noted at lines 405-408.

- From a logistical standpoint, conducting such experiments in tropical environments presents additional challenges. Baited field experiments in the tropics are susceptible to intense non-avian predation (e.g. ants/spiders/molluscs), which would vastly increase the number of censored (and therefore, incomplete) data points. This can obscure or limit the ability to measure avian predation, making a like-for-like repeat of the experiment in Panama infeasible.

It is also worth noting that similar field-based predation experiments have been widely used to test the adaptive significance of protective colouration in the literature. For example, Costello *et al.* (2020, *Proc R Soc B*) used a UK-based predation experiment to assess the function of false holes in leaf mimicry - a trait associated with tropical species, as noted in our manuscript (line 245). Similarly, Kjernsmo *et al.* (2020, *Curr Biol*) used non-native jewel beetle elytra as stimuli to test the camouflage role of iridescence under UK field conditions (now cited at lines 87-89). These studies, among others, demonstrate that the use of naïve predator communities is a well-established and effective method for isolating the functional significance of camouflage traits.

However, we appreciate that this rationale may not be immediately intuitive to readers, and we acknowledge that our original submission did not sufficiently elaborate on this point. To remedy this, we have now added the following clarification to the main text:

"To minimise potential effects of predator learning through search image formation - a phenomenon known to improve foraging efficiency in birds [14-17] - we deliberately performed the experiment in a locality where avian predators are naïve to leaf-masquerading insects. This approach, which mimics previous studies testing the adaptive value of protective colouration in tropical species using naïve predators in UK field environments [18,19], recreates the ecological conditions under which leaf masquerade first conferred a selective advantage, allowing us to isolate its functional properties independently of learned recognition. Since masquerade relies on the implicit assumption that prey are misclassified as their models, performing this experiment in a community of 'inexperienced' predators that do not routinely encounter leaf-masquerading prey enabled us to better ensure a leaf-like prey object would be classified as unprofitable by an insectivorous bird." (lines 84-95)

We also expanded on this point more fully in the methods:

"Conducting this experiment in a locality where avian predators do not routinely encounter leaf-masquerading katydids overcomes potentially confounding effects of prior experience, which could otherwise enhance predation on leaf-like stimuli (e.g. 'green ovals') if predators had learned they were profitable (see main text for details). Although our UK field site contains different passerine bird species to those found in Panama where katydids were sampled, conserved mechanisms of colour perception and spatial resolution across passerines mean that it is unlikely our targets would be perceived any differently by these species [58-60]." (lines 401-408)

- *>> Figure 1: how come the oval green trial doesn't have confidence intervals? What does "relative to the 'green oval' treatment" mean. Even if you scale it, why not the confidence intervals. Should explain in the legend or methods. The sample size is very high (n= 1296).*

Apologies for the confusion. Figure 1C plots the odds ratios derived from a linear mixed effects Cox survival model (using the parameter estimates and standard errors) where all treatment groups are compared relative to the 'green oval' treatment (i.e. 'perfect' leaf mimics). In Cox models, the hazard ratio for this reference category is implicitly set to 1. Because it is not estimated, the variance of the reference is undefined, but this serves as the baseline against which differences between the other treatments are calculated. As such, it is standard practice not to display error bars for the reference group in plots of this kind, as seen in other published work (e.g. see figures within Kjernsmo *et al.* 2020, *Curr Biol*; McLellan *et al.* 2021, *Curr Biol*; Rowe *et al.* 2021, *Proc R Soc* B; Wainwright *et al.* 2020, *Proc R Soc B*). To clarify this, we added the following sentence to the figure legend and cite some of the references above:

"As the reference category in the model, the hazard ratio for the 'green oval' treatment is fixed at 1 by definition and therefore has no associated confidence intervals [19,43]." (lines 279-281)

- *>> Not sure I agree with the statement "Therefore, shifts in color and pigmentation do not offer any obvious additional protective value other than the purpose of leaf masquerade."*

We understand why this phrasing might appear overly conclusive. We have rephrased it to make it more specific to habitat background related effects:

“Therefore, evolutionary shifts in colour and pigmentation do not appear to be driven by habitat-specific background matching.” (lines 129-130)

- *Concerns about the human scoring of leafiness:*

  >> The use of human participants to score leafiness is innovative, whose rationale is based on conserved mechanisms of object recognition between birds and primates. However, the Authors do not tell us what kind of advance this represents over previous methodologies or how it overcame previous methodological limitations.

As the reviewer notes, our rationale for using humans to score ‘leafiness’ was partly based on conserved mechanisms of object recognition between birds and primates but also because many primate species are known predators of tropical katydids. Previously, leaf masquerade in katydids had been categorised using a simple morphological metric (tegmen-to-thorax height ratio; Mugleston *et al.* 2016, *Inv Sys*) while leaf masquerade classifications in butterflies have focussed exclusively on wing pattern features specific to nymphalid Lepidoptera, which are not transferrable to katydid tegmina (Suzuki *et al.* 2014, *BMC Evol Biol*). Our method, by contrast, offers a holistic estimate of how leaf masquerade is perceived without relying on any one component trait (e.g. shape or colour) that underlies masquerade.

This design enabled us to study how variation in colour and shape interact to influence overall perceptions of leaf masquerade – a central aim of the study. To clarify this rationale, we have added the following to the main text:

“Unlike previous categorisations of leaf masquerade that relied on specific morphological features [4,11], scoring ‘leafiness’ in this way provides a holistic estimate of how leaf masquerade is perceived, without relying on any one component trait.” (lines 141-144)

And we have added the following sentence to the methods:

“These holistic measures of ‘leafiness’ allowed us to study whether variation in colour and shape interact to influence human perception of leaf masquerade.” (lines 487-489)

REVIEWER 3

- *I have carefully reviewed the manuscript "Extraordinary adaptations: Functional and evolutionary synergy of trait components can explain the existence of leaf masquerade" (PBIOLOGY-D-25-01133_R1). While the study offers valuable insights into the evolution of leaf masquerade in katydids and addresses a significant question in evolutionary biology, I must recommend rejection in its current form due to several major concerns that significantly impact the validity and conclusiveness of the findings.*

We regret that a lack of clarity in our original framing may have contributed to this reviewer’s concerns. However, the majority of these comments appear to stem from a misappreciation of very well-established methodological approaches that use digital photography to study animal colouration. Our responses are supported by the methodologies of a substantial body of published studies, including those cited in our detailed responses below. We therefore remain confident that the validity and conclusiveness of our findings are robust and have further clarified this through some additional analyses and edits to the main text and methods.

- *Major Comments:*

  1. Behavioral Experiment Design Issues:

  The experimental setup combining mealworms with colored paper targets (Lines 73-76, 216-227, Figure 1, Lines 273-275) raises concerns about ecological validity. Directly exposing mealworms may lead to unintended associations with other prey types, such as butterflies, potentially influencing predator behavior and compromising the conclusions. Additionally, the contrast between the colors and shapes of the paper targets and the mealworms themselves may introduce confounding variables. The authors do not adequately address how these elements interact from the predators' perspective. For guidance on experimental design, the authors may refer to a similar study conducted on orchid mantises by James C. O'Hanlon (2013, Journal of Orthoptera Research), which utilized quantified color (spectral reflectance) and shapes with artificial mantises.

This is a key point: our artificial targets were designed so as to *not* resemble any specific prey type. The objective was to extract general principles about the interaction of shape and colour that comprise leafiness, rather than cause a predator to misclassify targets as a particular insect. The models were designed to resemble leaf-like colours and shapes commonly found at our UK field site, so they could be plausibly misclassified by insectivorous predators as unprofitable objects. Because mealworms were exposed equally across all treatments, their detectability should not have differentially influenced the way targets were classified. We have already addressed the related concern about colour contrast of mealworms above (see pages 5-6).

We added the following clarification of our target design aims to the Methods section:

"Our aim was to create generic, ecologically relevant targets that could be perceived as leaf-like by wild passerine predators, without resembling the morphology any specific local insect prey species." (lines 334-336)

We are grateful to the reviewer for bringing additional literature to our attention. O'Hanlon (2013) used spectrometry to quantify colour variation in orchid mantises and modelled these data to an ecologically relevant hymenopteran visual system – a highly appropriate approach for studying UV-reflecting prey that interact with UV-sensitive insects. However, in our case, such methods are not warranted. While the wings of orchid mantises possess UV-reflecting patches, the surfaces of tree bark and plant leaves, which our targets were designed to resemble, reflect little to no UV (see Cuthill *et al.* 2006, *Proc R Soc B*; Grant *et al.* 2003, *Agr For Met*; Woolley, 1971, *Plant Phys*). As a result, calibrated digital photography has become a standard and widely used technique in creating targets for similar experiments, particularly within the last 10 years (e.g. see Barnett *et al.* 2018, *R Soc Open Sci*; McLellan *et al.* 2021, *Curr Biol*; Michalis *et al.* 2017, *Proc R Soc B*; Wainwright *et al.* 2020, *Proc R Soc B*), due in large part to advances in image processing and visual modelling techniques (see Troscianko & Stevens, 2015, *Methods Ecol Evol;* van den Berg *et al.* 2019, *Methods Ecol Evol*).

Therefore, by converting raw multispectral images into blue tit cone catch quanta and using them to calculate mean RGB values for our target treatments, we ensured that the reflectance of our targets fell within variation observed in the natural background objects they were designed to resemble, as viewed by ecologically relevant predators.

We added the following clarifications to the methods and refer to the references above:

“Because leaves (and tree bark, see below) reflect little to no ultraviolet (UV) light [47,48], standard digital photography was used to create ecologically relevant target stimuli following established approaches [e.g. 18,43,47,49-54]. (lines 337-339)

“…within the MICA toolbox, a widely used software attachment for conducting image analysis from the perspective of different animals’ visual systems [52,55].” (lines 347-349)

“The reflectance of our green and brown targets therefore fell within variation observed in the leaves and bark they were designed to resemble.” (lines 358-360)

- *2. Intraspecific Variation Overlooked:*

  Many masquerading species, such as stick insects, exhibit intraspecific diversity in colors, shapes, and sizes. This phenomenon is well-documented in orchid mantises (James H. et al., 2013-2016; Xin Zhao et al., 2023-2024). Similarly, katydids show significant variation in color and shape within the same species and even within the same population. By examining only one morph per species, the authors overlook this critical source of variation, which is essential for understanding the evolutionary dynamics of these traits. This omission weakens the conclusion that color and shape have co-evolved synergistically.

Apologies for not clarifying this point in our original submission. While some katydid species do exhibit notable within-species variation in tegmen colour (see an extensive discussion of this in relation to a comment by reviewer 1 on pages 2-3), all species included in our phylogenetic comparative analysis were monomorphic for the traits we measured at our sampling locality. Specifically, all individuals sampled from green and/or pigmented species were consistently green and/or pigmented with the same being true for species that were brown and/or non-pigmented. To address this, we have added the following to the methods:

“All individuals of the sampled species were monomorphic for the categorical colouration states we obtained, meaning that colour classifications based on a single individual were representative of the species as a whole at our sampling locality.” (lines 450-453)

Subtle intraspecific variation in tegmen aspect ratio does exist in our studied species, but our original MCMCglmm analysis on evolutionary associations between colouration and shape used individual-level data (N = 288) and therefore accounted for within-species variation. Between-species variation was also accounted for by including species as a random effect. While only a single representative individual was shown to participants during the online survey, our analyses of ‘leafiness’ scores used species mean aspect ratio values from all sampled individuals. Thus, any intraspecific variation in shape does not affect our overall argument that shape and colouration interact non-additively to influence human perceptions of ‘leafiness’ in katydids.

To further account for intraspecific variation, we reran our shape~colouration MCMCglmm model, including sex and its interaction with green pigmentation as additional fixed effects. While a significant effect of sex was detected, the interaction between sex and green pigmentation was not significant. Importantly, including sex did not alter the evolutionary association between tegmen aspect ratio and green pigmentation. To address this change, we added the following to the main text:

“This model also revealed that female tegmina exhibit significantly lower aspect ratios compared to males (P-mean = -0.014, 95% CI = -0.023 – -0.005, P_{MCMC} = 0.002), but the interaction between green pigmentation and sex was not significant (P-mean = -0.002, 95% CI = -0.016 – 0.012, P_{MCMC} = 0.847).” (lines 205-208)

And added the following detail to the methods:

"In these models, sex and its interaction with colouration were included as additional fixed effects and species was included as a random effect to control for between species variation." (lines 528-530)

We agree that the existence and adaptive significance of within-species polymorphism in katydids is a fascinating area for future research, and we modified the text to address this in response to a comment made by reviewer 1 (see page 3).

- *3. Flawed Predator Vision Modeling:*

  The study's reliance on human visual assessments is problematic, as it cannot be directly applied to the visual systems of actual predators like birds. Birds possess tetrachromatic vision, allowing them to perceive ultraviolet reflectance, a capability humans lack. Converting RGB camera data to simulate avian vision is not a suitable substitute for directly modeling the spectral sensitivities of avian visual systems. Furthermore, while the authors mention that some New World primates are dichromatic and may have different visual capabilities (Lines 142-144, 162-165), using trichromatic humans as proxies for predators is questionable and requires stronger justification. Given that predator visual perception is central to the study's hypotheses and conclusions, this issue is particularly critical.

We disagree with the rationale underlying this criticism. We do not 'directly apply human visual assessments to avian visual systems'. We apologise if our original manuscript gave this impression to at least one reader; hence we have made changes (detailed below) to avoid risk of any reader misunderstanding the rationale behind our use of humans to score leafiness. A central aim of our human-based online survey was to test if the functional interaction between colouration and shape, already observed in our avian predation experiment, was concordant with human perceptions of leafiness (as stated on lines 161-163 of the main text). Many trichromat primates, such as tamarin monkeys which are locally present at our Panama field site, are known katydid predators (Smith *et al.* 2012, *Anim Behav*) so understanding how leaf masquerade is perceived by primate visual systems is also of ecological relevance. The conclusions derived from these analyses were informed by both established work on object recognition in birds and primates (Gibson *et al.* 2007, *Curr Biol*; Nielsen & Rainer, 2007, *Curr Biol*; Soto & Wasserman, 2014, *Front Neur Circ;* cited on line 165), and by our field predation experiment using avian predators. Together, these lines of evidence support the inference that bird and primate visual systems perceive leaf masquerade in broadly comparable ways. In summary, our approach integrates experimental and previous comparative data to arrive at these conclusions, rather than assuming equivalence between human and avian vision from the human survey data alone.

As noted previously (see response on pages 13-14), using digital photography to calibrate RGB camera data to avian cone catch is a well-established method in visual ecology. We stress that this calibration was only performed when designing the targets used in our avian field predation experiment. In contrast, the katydid lightbox images shown to human participants were non-linearized JPEGs that included an XRite colour chart to maintain colour constancy. This is now explicitly highlighted in the methods as follows:

"…a randomly selected light box JPEG image (non-linearized, with a ColorChecker passport included) of each of the 51 katydid species…" (lines 477-478)

Although our original submission did not consider potential ultraviolet reflectance in katydid tegmina, this omission has no bearing on our non-UV-sensitive human-based metrics of leafiness. Therefore, any UV cues would not have influenced how katydid 'leafiness' was

scored by humans – nor would it have influenced perception by dichromatic primates which are also non-UV-sensitive. Nevertheless, to address this point more thoroughly, we scanned a random subsample of katydid tegmina from our dataset using a spectrophotometer at a wavelength range of 290-700 nm and a resolution of 1 nm. All scanned tegmina showed a consistent reflectance distribution which revealed no notable UV reflectance, confirming that it is unlikely ultraviolet-sensitive predators (i.e. birds) use UV cues when detecting these katydids. See the figure and legend below, which we now include in the Supplementary Materials as Figure S3:

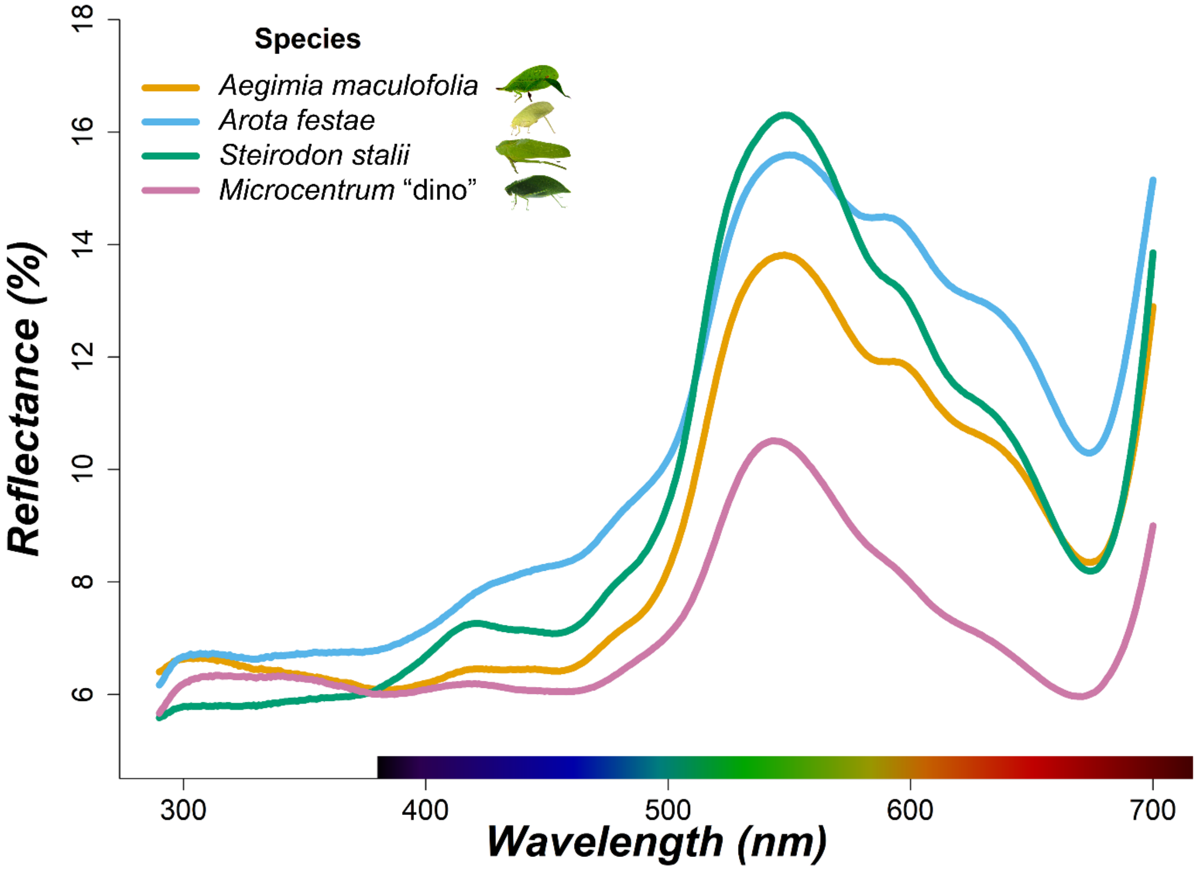

*"**Figure S3.** Mean reflectance values for the tegmina of four randomly katydid species within a 290 to 700 nm spectral range (n = 2 per species)."* (lines 772-774)

We have also added the following clarification to the main text:

"Spectrophotometry from a random subset of tegmina confirmed minimal ultraviolet (UV) reflectance, indicating that the appearance of the artificial targets used in the avian predation experiment are comparable to real katydid tegmina, in that both reflect minimal UV (Figure S3; see Materials and methods)." (lines 124-127)

And the following to the Methods:

"To confirm that katydid tegmina show minimal reflectance outside the visible range, reflectance was also measured from a random subset of eight unmanipulated katydid tegmina (four species) by mounting specimens adjacent to an integrating sphere and scanning with a V-770 spectrophotometer (JASCO, Tokyo, Japan) (wavelength range: 290-700 nm, resolution: 1 nm), calibrated using a white reflectance standard." (lines 456-461)

While this additional spectral analysis validates the comparability between the artificial targets used in our predation experiment and the tegmina of real katydids used in our comparative analyses (i.e. both had minimal UV reflectance), it is almost certainly not relevant to the context of the binary colouration data (green vs. brown) that we collected from our tegmina samples. The results of our avian field predation experiment provide direct evidence that green *vs.* brown colouration is under strong selection by insectivorous tetrachromatic birds to enhance masquerade. The strong concordance between this predation experiment and our human assessments further implies that the functional interaction between colouration and shape in enhancing masquerade is broadly shared across visually guided animals.

In response to the point about dichromacy, although seemingly unambiguous differences in colour between green and brown katydid tegmina may appear less nuanced to dichromats, it is important to note that many New World primates are polymorphic di-/trichromats. Notably, dichromat morphs have been shown to outperform trichromats in detecting camouflaged prey (Melin *et al.* 2007, *Anim Behav;* Troscianko *et al.* 2017, *Behav Ecol*). Therefore, using human trichromats to assess leafiness provides a perhaps conservative estimate of how more ecologically relevant predators perceive camouflaged objects. It is also worth noting that the use of humans as proxies for how more ecologically relevant primates detect camouflaged items is a method validated by numerous existing studies (e.g. Kjernsmo *et al.* 2020, *Curr* Biol; Matchette *et al.* 2018, *Anim Behav;* Troscianko *et al.* 2021, *Comm Biol*; Troscianko *et al.* 2013, *PLoS One*).

We provide further clarifications regarding our rationale behind the use of the human online survey in response to a previous reviewer comment (see page 12). We have also rephrased the following sentence which better highlights the potential ecological relevance of our human-based assessments:

"While many New World primate predators of katydids are also trichromats, thus enhancing the ecological relevance of our human-based assessments of leafiness, these metrics might represent a conservative estimate of how colour and shape interact in the eyes of dichromatic species, whose visual systems are thought to improve the ability to find camouflaged prey [27,31,32]." (lines 184-188)

And have added the following to the methods:

"Our use of humans as proxies for understanding how more ecologically relevant primates detect camouflaged items has been validated by an extensive body of prior work [19,43,65-67]." (lines 489-491)

- *Due to these significant concerns, I believe the manuscript requires major revisions that go beyond the scope of typical revisions. These issues affect the core validity and interpretation of the study. Therefore, I recommend rejection of the manuscript in its current form. However, I encourage the authors to thoroughly revise their manuscript, addressing the experimental design issues, incorporating intraspecific variation and sexual dimorphism into their analysis, and refining their approach to predator vision modeling. A revised manuscript that adequately addresses these concerns could potentially be suitable for reconsideration.*

We hope our above clarifications have alleviated these concerns.

- *In addition, I suggest the authors use the term "mimicry" precisely. Masquerade strategies differ fundamentally from mimicry strategies in that masquerading species do not mimic specific "target" objects. For example, it is often impossible to specify exactly which plant species a stick insect is mimicking.*

We did not use the term “mimicry” anywhere in our original manuscript, although we did use “leaf-mimicking”. For clarity, we have replaced all mentions of “leaf-mimicking” with “leaf-masquerading”.

- *Key references:*
  O'Hanlon JC. 2014. The Roles of Colour and Shape in Pollinator Deception in the Orchid Mantis Hymenopus coronatus. Ethology 120:652-661.
  Smith AC, Surridge AK, Prescott MJ, Osorio D, Mundy NI, Buchanan-Smith HM. 2012. Effect of colour vision status on insect prey capture efficiency of captive and wild tamarins (Saguinus spp.). Animal Behaviour 83:479-486.
  Zhao X, Liu J-X, Chen Z. 2023. The orchid mantis exhibits high ontogenetic colouration variety and intersexual life history differences. Evolutionary Ecology 37:569-582.

We thank the reviewer for bringing this interesting literature to our attention.

- O’Hanlon (2014) – now cited at line 226.
- Smith *et al.* (2012) – cited in our original MS, now lines 164 and 188.
- Zhao *et al.* (2023) collected observational data on a species of orchid mantis. They show that aggressive mimicry in this species is not hostplant specific and is characterised by striking colour polymorphism, amongst other interesting aspects of its natural history. While some of these findings are analogous to observations made in Tettigoniidae (e.g. apparent lack of hostplant specificity; Palmer *et al.* 2022, *Diversity*), we feel that the focus of this study is too taxon-specific to be directly relevant to the questions addressed in our manuscript.

REVIEWER 4

- *This is a well written manuscript where the authors assess both the antipredator effects of "leafiness" and the evolution of the composite traits that confer leafiness to katydid tegmen. I appreciate the integration of a field model predation study, a human tegmina pattern assessment study, and the evolutionary model. I think this work complements the genomic and gene manipulation methods used to assess the evolution of leaf mimicry in butterflies, and provides new insights. I have a few questions concerning why the authors categorized models that made it to the end of the predation study without being attacked or lost as "censored" and similar to those that were lost, as well as some clarifying questions concerning method detail in the main text and whether katydid age or sex may influence the "leafiness" assessment in the evolutionary models. See below.*

We are grateful for these favourable comments and appreciation of our combination of approaches and analytical methods.

- *Lines 80-93: It would be useful to know how many of the artificial wing targets were attacked, to get a sense of the effect sizes reported here. Also, some information concerning what the artificial wing targets were would be helpful, since the methods section comes after the results. Were these artificial wings pinned to worms? Or to clay? How was survival assessed?*

  For this short form type of manuscript it would be incredibly helpful for the reader to have some of these important methodological details (as well as the actual

percentage of models attacked) mentioned in the results. Even though some of this information is in Figure 1, it would be useful to also mention this information in the main text.

We agree that reiterating these methodological details in the main text would help readers more easily interpret our results, given the methods-section-last format of the manuscript. We therefore added the following to the main text:

"Survival of each individual target was assessed based on the presence or absence of an edible mealworm bait, which served as the 'body' of the target." (lines 83-84)

We also now quote the total number and percentage of targets that were predated, and in response to a comment below, the percentage of targets subject to non-avian predation, lost, and survived. This line of the results now reads:

"Overall, 39.6% of targets (513) were attacked by birds (51.9% showed signs of non-avian predation, 1.9% were lost, 6.3% survived until the end of the trial)." (lines 95-96)

- *Line 89: sample sizes should be included with these statistical outputs, especially since the results are reported before the methods. They should be in the main text as well as in the figure legends.*

We thank the reviewer for pointing out this omission. We added sample sizes to the statistical outputs, and this section now reads:

"The interaction between colour and shape predicted survival rate (mixed-model Cox regression: $\chi^2_4 = 9.882$, N = 1296, p = 0.043), but neither factor was significant as a main effect (mixed-model Cox regression: colour, $\chi^2_2 = 2.147$, N = 1296, p = 0.342; shape, $\chi^2_2 = 5.878$, N = 1296, p = 0.053). Furthermore, colour affected survival rate only for oval-shaped targets (mixed-model Cox regression: oval, $\chi^2_2 = 7.143$, N = 432, p = 0.028; elongated oval, $\chi^2_2 = 2.058$, N = 432, p = 0.357; rectangle, $\chi^2_2 = 2.814$, N = 432, p = 0.245; Figure 1C; see Table S1 for post-hoc tests)." (lines 99-105)

Please note that sample sizes were already included in the Figure 1 legend (line 277).

- *Figure 2: In general, this is a highly informative figure. However, it is difficult to differentiate between green + pigmented and the top tegmen on the not green + not pigmented side. Perhaps the authors could include larger images for this comparison in the supplemental file, so the differences between pigmented and non-pigmented tegmen are easier to detect?*

We are grateful for this suggestion. Instead of including an additional supplementary figure, we replaced the original images in Figure 2B (which were light box photographs of unmanipulated tegmina) with images of tegmina between microscope slides (see methods, lines 462-469). This provides a much clearer contrast between the different colouration states. The updated version of Figure 2B can be found on page 7, as part of a response to a comment made by reviewer 1.

- *Line 257: Is there a way to age these katydids? And, does tegmen coloration or shape change with age? In addition, are tegmina sexually dimorphic? The authors do mention that they are used during mate selection for some species. The authors say they used 3 per species, but do not mention sex. Some discussion of sexual dimorphism (or lack thereof) should be included here.*

All sampled katydids were adults. However, to our knowledge, there is no reliable method for estimating the age of wild-caught katydids beyond their final moult. While some species exhibit notable within-species variation in colour and shape, it remains unclear whether such variation is age-related. We address related points concerning polymorphisms on pages 2-3 and 14-15.

Regarding sexual dimorphism, male katydids - like all ensiferan insects – bear stridulatory structures on their tegmina that produce acoustic signals for mate attraction. Unlike the rest of the tegmen which naturally rests laterally (in the Panamanian species we sampled) along the body and contributes to leaf masquerade, the stridulatory area is positioned perpendicularly on the dorsal surface and is therefore functionally and physically distinct from the area involved in leaf masquerade. As noted in the methods (lines 471-472), the shape of the stridulatory area was excluded from tegmen aspect ratio measurements. Nonetheless, we now account for intersexual variation in our analyses, as described in an earlier response on pages 14-15.

- *Lines 350-351: Why would targets that survived for the full 72 hours be considered 'censored'? Shouldn't that set just be considered non-predated? I understand the authors' justification for the non-avian predation, being lost, or relocated (though all these outcomes should be reported), but I don't understand why those that still had mealworms at the end of 72 hours and were attached to the three should be considered 'censored'. Please clarify.*

To clarify the rationale behind this aspect of the analysis: in the context of survival analyses, censored data are not missing data, but incomplete observations. Specifically, when a target survives predation until the end of the 72-hour testing period, we know that the event (predation) did not occur during the observation window, but we do not know whether, or when, it would have occurred had the experiment continued. This approach allows us to incorporate all available data without biasing estimates of relative survival or hazard ratios by excluding targets that were not predated. We understand why the wording might be confusing, so we added the following to the methods to explain this to other readers:

"In survival analysis, censored data are incomplete observations where the exact time of an event (i.e. predation) is unknown, but it is known that the target survived until a certain point. These data allow us to incorporate all available information – from both complete and incomplete records of predation events – into analyses." (lines 427-430)

As requested, we also now report the total number and percentage of targets that were lost or experienced non-avian predation. These are quoted on page 19 in response to a previous comment by this reviewer.

- *Line 609: should be a space between "the" and "calibrated"*

This sentence has now been corrected.

- *Figure S1: Please include confidence interval shading on this survival plot.*

Certainly. Figure S2 (formerly Figure S1) now appears as follows:

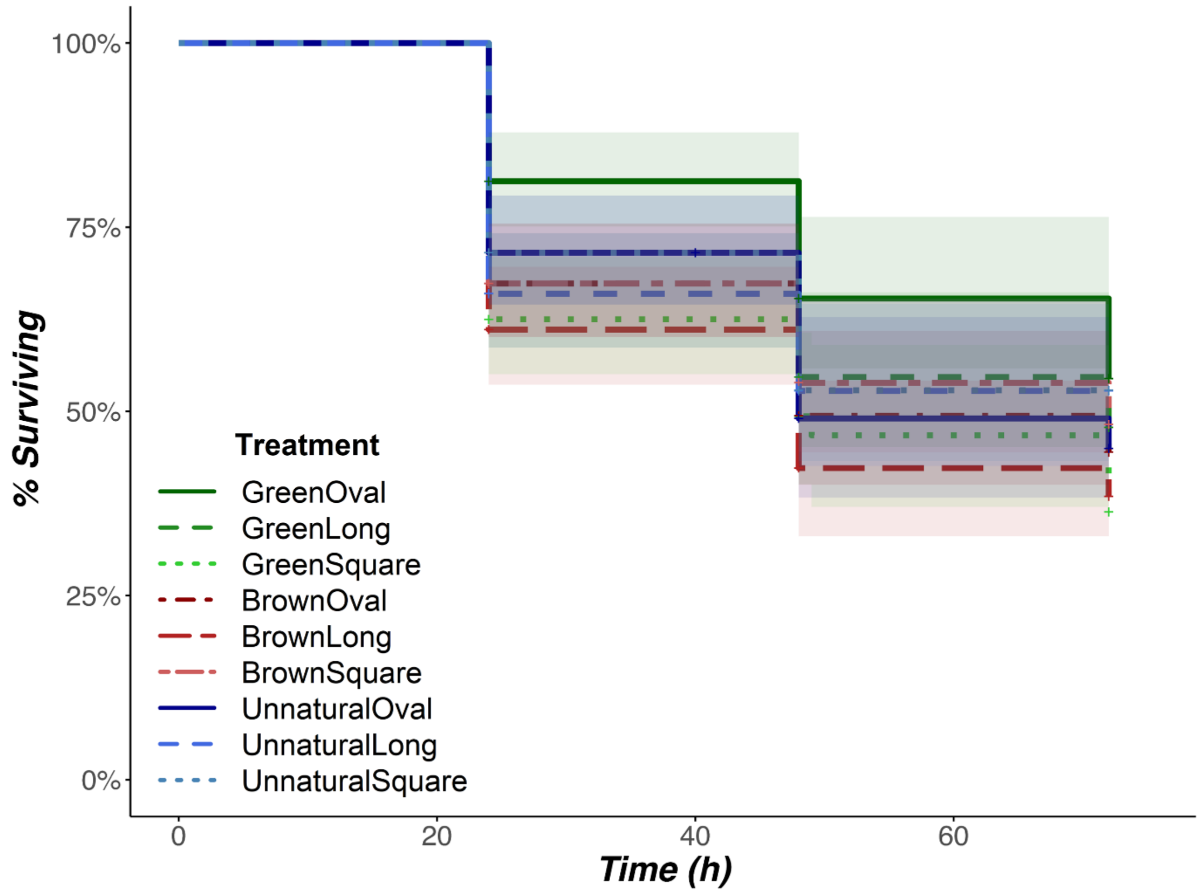

And have added the following to the legend:

"Shading represents 95% confidence intervals for each treatment." (lines 768-771)

- *Figure S2: Please include description of what the proportions of green and grey at the nodes indicate in the figure legend.*

Apologies for the lack of clarity. We have now added the following sentence to the Figure S4 (formerly Figure S2) legend:

"Pies at each node represent the posterior probability of the ancestor having each character state." (lines 779-780)
