# Supplementary material for: Functional and evolutionary synergy of trait components can explain the existence of leaf masquerade in katydids
Source: PLoS Biol. 2025 Nov 3;23(11):e3003468. doi: 10.1371/journal.pbio.3003468 (PMC12582443; doi:10.1371/journal.pbio.3003468)
Supplement: Attachment — Submitted filename: Response to Referees.docx [file pbio.3003468.s004.docx]

**Response to Referees**

Below we provide information on how we have addressed the editor and reviewers’ comments, including all editorial changes to the text.

Original reviewer comments are in black italics, the authors’ responses in blue, and quotes from corrected text in blue italics. Line numbers in brackets refer to the updated manuscript, without inclusion of tracked changes.

**EDITIOR COMMENTS**

- *“a) We try to avoid punctuation in our Titles. Please change your Title to "Functional and evolutionary synergy of trait components can explain the existence of leaf masquerade in katydids"”*

Certainly. We have changed the title, so it now reads as requested:

*“Functional and evolutionary synergy of trait components can explain the existence of leaf masquerade in katydids”* (lines 2-3)

- *“b) Please attend to the remaining comments from the reviewers. I asked the Academic Editor about reviewer#3's requests, some of which might involve additional data or analysis; they said, "It to the credit of reviewer #3 to have made such effort to scrutinise the paper. I don't see however any of the suggestions to be critical. These are all manners to verify details. I would give the authors the option to address comments if they already have the data. If they choose not to, they they can fix the edits and move on." I hope that this guidance is helpful.”*

We are very grateful to the academic editor for providing this additional guidance. We agree that none of reviewer 3’s suggestions are critical, and indeed, some of these details were already included in the submitted manuscript. We have addressed the remaining suggestions as thoroughly as possible.

- *“c) Please address my Data Policy requests below; specifically, we need you to supply the numerical values underlying Figs 1C, 2ABCDE , S1-S5, either as a supplementary data file or as a permanent DOI’d deposition. I note that you already have an associated Zenodo deposition, for which you provide a tiny.url reviewer link. Please could you confirm whether the data and code in this deposition are sufficient to recreate the Figures? If not, please include it in your deposition or as a supplementary file(s).”*

We confirm that the data and code within the Zenodo deposition are sufficient to recreate all figures.

- *“d) Please cite the location of the data clearly in all relevant main and supplementary Figure legends, e.g. “The data underlying this Figure can be found in S1 Data” or “The data underlying this Figure can be found in* [*https://zenodo.org/records/XXXXXXXX*](https://zenodo.org/records/XXXXXXXX)*”*

We confirm that the location of the data has now been cited in all figure legends, using the suggested notation.

**NB.** We also confirm that we have addressed all technical requests and formatting changes.

**REVIEWER 1**

- *“I have read the new version of this manuscript and the response of the authors to reviewers, and I should say I am satisfied with the changes and clarifications. I believe that this version has improved content readability, the figures are of good publication standards, therefore I have recommended acceptance.”*

We are pleased to have addressed all of this reviewer’s comments appropriately and thank them for the recommendation.

**REVIEWER 2**

- *“In this revised version, the Authors have done a good job at responding to most of the critiques I raised from the first round of review. Therefore, I think this manuscript is worthy of publication in PloS Biology. I just have one final comment / critique that I hope that the Authors can address prior to publication, which I believe would clarify the argument the Authors are trying to make.”*

Likewise, we are pleased that we addressed this reviewer’s critiques and thank them for the recommendation.

- *“The Authors propose two alternative hypotheses: "non-adaptive stepwise evolution" versus "Adaptive synergistic evolution" of complex composite traits. However, I remain unclear about what the Authors mean by "Non-adaptive." I believe what the Authors mean is non-adaptive with respect only to leaf masquerading. If so, the Authors should state this clearly. Otherwise, general readers would take these shape and size traits to have no function with respect to the organism, and as such, would evolve neutrally and eventually be lost. I don't think this is what the Authors mean. It is more plausible that these traits serve alternative functions, and where therefore retained by selection for other functions unrelated to leaf-masquerading, and then were co-opted via directional selection to form composite trait with adaptive value for leaf masquerading. The Authors would do well to cite and refer to Vrba and Gould's classic paper on co-opiton in morphological evolution. Basically, what I have described here is co-option from a trait that previously has a different function, what they call "aptation" versus co-option of a trait that previously had no function "non-aptation."

  I feel that maintaining and clarifying this distinction between aptation and non-aptation (the Authors don't have to use this exact terminology as it is not frequently used, just describe it) would help readers understand the broad evolutionary significance of the results of this manuscript, and would help readers better understand the what the two alternative scenarios actually mean for the evolution of complex adaptations.”*

We agree that it is worth clarifying this point to avoid potential confusion. This reviewer is indeed correct that we mean “non-adaptive” only with respect to leaf masquerade and that the ideas discussed by Gould & Vrba (1982) are appropriate in this context. To address this, we have rephrased the following abstract sentence as follows:

*“When these component traits do not enhance fitness independently of one another, their origin requires that they evolve sequentially through intermediate steps that do not produce their full adaptive value as a combined trait, or alternatively, that they arise via simultaneous, synergistic evolution.”* (lines 20-23)

We have also rephrased the following sentences of the introduction, citing Gould & Vrba (reference 6 below):

*“The evolutionary origin of such extreme adaptive complexity has been a persisting question since the birth of evolutionary biology, particularly when component traits do not appear to increase fitness additively [4,5]. For the springboard prey-trapping mechanism of Nepenthes carnivorous pitcher plants, it has recently been demonstrated that component traits evolved independently, likely whilst under selection for other functions, implying that complex composite adaptations can arise through intermediate evolutionary stages unrelated to their value as composite traits, and later be co-opted for novel functions [3,6].”* (lines 38-44)

And – borrowing with thanks (see Acknowledgements, lines 581-582) to the reviewer’s helpful phrasing – we added the following to the results/discussion:

*“**In other words, were colouration and shape selected independently at different timepoints, one maintained by selection for functions unrelated to leaf masquerade and later co-opted to form a composite masquerade trait with the other? Or did they both evolve simultaneously and result in this form of camouflage? Non-adaptive stepwise evolution would imply no evolutionary correlation between colouration and shape across the phylogeny – that is, these traits would not tend to arise at the same time during the evolutionary history of these species because neither would enhance masquerade in isolation.”* (lines 198-204)

**REVIEWER 3**

- *“I have carefully assessed the authors' responses to my comments and those of the other reviewers, as well as the overall quality of the revised manuscript.

  First, regarding my original concerns, the authors have addressed them comprehensively. For the issue of behavioral experiment design, they clarified that the goal of the experiment was to extract general principles of color-shape interaction rather than mimic specific prey, confirmed through bird-calibrated images that mealworms do not interfere with target classification, and reasonably explained why spectrometry (as suggested in O'Hanlon 2013) was unnecessary due to minimal UV reflection from leaves and bark. On intraspecific variation, they demonstrated that sampled species were monomorphic for measured traits at the study site, used individual-level data and species as a random effect to account for shape variation, and further incorporated sex into analyses—finding no impact on the core evolutionary association between color and shape. For predator vision modeling, they clarified the purpose of human leafiness scoring (to align with avian predation results, not directly substitute avian vision), confirmed minimal UV reflection in katydid tegmina via spectrophotometry, and cited literature to validate humans as proxies for ecologically relevant primates.

  These changes have elevated the manuscript from "major revision/rejection" to "minor revision". Two small, easily addressable issues remain:”*

We are pleased that our justifications were clear and that our revisions have made our approach more accessible to readers less familiar with these methods.

- *“1. A supplementary control (or at least discussion) eliminating the olfactory/visual confound of the exposed mealworm bait.”*

There is no confound: All models had mealworm baits, so any olfactory cues emitted by these mealworms would have been consistent across all treatments as we now emphasise in the Methods:

*“All targets used mealworm baits, eliminating the potential for experimental confounds across treatments.”* (lines 348-349)

As reviewer 4 correctly states (see page 5), using mealworms as bait is standard practice for these types of experiment (e.g. see Costello *et al.* 2020, *Proc R Soc B;* Cuthill *et al.* 2005, *Nature*; Stevens *et al.* 2007, *Anim Behav;* Yeager *et al.* 2024, *Ecol & Evol*).

- *“2. A brief tetrachromatic visual‐modelling check on a subset of katydid tegmina to confirm that UV contrast is irrelevant (or provide ΔS values if it is).

  Both can be handled with additional supplementary material and do not require new field work.”*

Although our sample size was too small to test this statistically, plotting the mean tegmen reflectance for four katydid species in blue tit tetrachromatic colour space confirms minimal photon catch by the UV-sensitive cone. We now present this as an inset to Figure S3 as follows:

And have added the following to the legend:

*“The inset plots these data in tetrachromatic avian colour space using the blue tit (Cyanistes caeruleus) visual model, where points represent the photon catch of the blue tit shortwave (S), mediumwave (M), longwave (L) and ultraviolet (UV) cones.”* (lines 791-796)

- *“Synthesizing comments from other reviewers and the revised manuscript's quality, there are still areas for improvement. In response to Reviewer 1's suggestion to include Mimetica and Typophyllum, while the authors' explanations (sampling challenges, polymorphism, uncertain phylogeny) are valid, future work could explore expanded sampling and specialized methods for these genera to enhance conclusion generality.”*

We previously noted on lines 266-268 the potential for such interesting work, and necessity of expanded sampling.

- *“For Reviewer 2's concern about phylogenetic path analysis, the authors could present detailed comparisons of alternative models (stepwise, simultaneous, hybrid) in supplementary materials (e.g., tables of path coefficients and fit statistics) to better illustrate why simultaneous evolution is favored.”*

This information, including fit statistics (i.e. AIC scores), were already provided in Figure S5 (lines 803-809).

- *“Addressing Reviewer 4's question on "censored data," the authors might add a brief supplementary analysis to show how excluding such data would affect results, reinforcing the robustness of their survival analysis.”*

Excluding censored data would reduce our sample size by 60.1% (see lines 97-99), leading to a substantial loss of statistical power. More importantly, rerunning the analyses on only uncensored observations is not standard practice in survival analysis (see quoted references in response to this reviewer’s comment on page 4). As we noted in our original response to reviewer 4, these censored data points include targets that survived until the end of the testing period so removing them would discard meaningful information and bias estimates of relative survival. For these reasons, we have not rerun these analyses on uncensored data.

- *“Additionally, some tables (e.g., Table S3, S4) could have clearer formatting (consistent decimal places, explicit parameter labels) to improve readability.”*

All supplementary tables already report values to a consistent three decimal places and adhere to our parameter labelling standards. Therefore, without more specific recommendations, we are unsure how these could be improved.

- *Overall, the authors have effectively resolved major concerns, and the revised manuscript demonstrates strong scientific rigor—integrating field experiments, phylogenetic analyses, and human perception assays to advance understanding of leaf masquerade evolution. The remaining improvements are minor and do not compromise the core conclusions. I therefore recommend a "Minor Revision" for this manuscript. With these final adjustments, the work will be a valuable contribution to the fields of evolutionary biology and camouflage research.”*

We thank this reviewer for their recommendation and positive comments about our study’s rigour and impact.

**REVIEWER 4**

- *“I think the authors did a good job addressing reviewer concerns, and enhancing their justification for their experimental design. While the methods the authors used are somewhat standard for predation experiments at this point (mealworms are commonly used), the additional clarification is useful for readers who are less familiar with this type of experimental design.*

*I also appreciate the authors' expanded justification for avoiding predators who have learned to identify leaf mimics, as predators are notoriously good at learning how to find food, and many tropical birds are long lived and highly likely to be experienced with leaf mimics.”*

We thank the reviewer for these kind remarks and appreciation of our methodological approach.

- *“Now that the authors have included sex in their analyses of tegmina shape, and have found an effect of sex on aspect ratio, I would like the authors to include sample sizes for sex by species in the methods/supplemental.”*

We now provide an additional supplementary table (Table S2) which reports the sample size for sex per species, alongside all other summary data (colouration classification, mean tegmen aspect ratio, leaf masquerade likelihood and mean leafiness score). It has the following legend:

*“****Table S2.*** *Summary data for all 58 sampled species on Barro Colorado Island, Panama (N = 288).”* (lines 815-816)

We now also quote the total number of sampled males and females in the results/discussion:

*“…we studied the tegmina of 288 wild-caught individuals of 58 sympatric katydid species (196 male, 92 female; see Table S2) from a diverse…”* (lines 120-121)

- *“Below are some additional comments, line numbers are for the mark up version:

  Lines 65 and 68: I disagree with Reviewer 3's insistence on not using the term "leaf-mimicking" here, as insects with wings/tagmina that look like leaves are generally called leaf mimics, and the phenomenon as leaf mimicry (see Wang et al., 2022, Cell, for a recent example).”*

We agree with this reviewer that the term “mimic” does not necessarily need to refer to the resemblance of specific organisms/objects. However, ‘leaf-masquerading’ is effectively synonymous in this context, so we have opted to keep this phrasing.

- *“Line 85: "at sampling" needs to be added here, otherwise the methods aren't clear. "Survival of each individual target at sampling was assessed based on the presence or absence of an edible mealworm bait, which served as the 'body' of the target.”*

This change has now been made:

*“Survival of each individual target at sampling was assessed…”* (line 85)

- *“Line 161: I'm not sure I'd agree with the authors that the OU model being the best fit suggests "strong" directional selection. Directional selection, sure, but the word "strong" is pretty vague here. I suggest the authors remove the term "strong" or provide additional justification for their use of the term.”*

This is a fair point. The word “strong” has now been removed.

- *“Line 216: Interesting to see that female tegmina have lower aspect ratios compared to males, perhaps the two sexes are mimicking different tree species? And, given that there was an effect of sex on aspect ratio, please be sure to include the sample sizes for sex.”*

To our knowledge, little is known about intersexual habitat differences in these species, and from personal observations, they do not seem to mimic specific plant species. Nevertheless, this result indeed suggests that tegmina shape is under different selection pressures in males and females, but we currently lack sufficient information to speculate what those pressures might be. Accordingly, we have added the following to this sentence:

*“This model also revealed that female tegmina exhibit significantly lower aspect ratios compared to males (P-mean = -0.014, 95% CI = -0.023 – -0.005, P_MCMC_ = 0.002), suggesting that males and females likely experience different selection pressures, but the interaction…”* (lines 212-214)

Given its Bayesian framework, it is not standard practice to report sample sizes alongside parameter estimates from MCMCglmm analyses. However, as noted in our response on page 6, sample sizes for sex are now reported elsewhere in the manuscript.

- *“Line 227: please state what the two (or three?) human-perceived metrics of leafiness are here, so readers don't have to remember. If it's all three, then it should be "any" instead of "either".”*

This change has been made, and the sentence now reads as follows:

*“When both leaf masquerade probability and mean leafiness score were used as the dependent variable, the path analysis yielded…”* (lines 223-224)

- *“Line 233: Here and earlier- this statement suggests that leafiness was never lost. Is that correct? That leaf masquerade has been gained multiple times in this system but never lost? Please clarify.”*

We agree that the original phrasing was confusing. Consistent with existing data (Mugleston *et al.* 2016, *Inv* Sys), our results support multiple secondary reductions in “leafiness” among our studied species, as noted on lines 160-162 and illustrated in Figure 2A. We have added “acquisition and loss” to these sentences, so they now read as follows:

*“…the path analysis yielded strong support for models of simultaneous acquisition and loss of all three traits, supporting their coordinated, synergistic evolution during the origin of leaf masquerade (Figure 2E; Figure S5; Table S7). Taken together, the evolution of leaf masquerade in our studied species is best explained by the simultaneous, synergistic acquisition and loss of trait components, under directional selection.”* (lines 224-228)

- *“Methods lines 489-494: Please include sample sizes for sex per species.”*

Table S2 (see our response on page 6) is now cited here.

- *“Lines 242-244: This is a good place to reiterate that females had different aspect ratios than males, further suggesting other selective forces acting on the trait.”*

We agree. We have now added the following sentence:

*“The latter might explain the sex differences in tegmen shape that we observed.”* (line 238)

- *“Figure 2: The pigment vs non-pigment is much easier to see in the revised figure.”*

We are pleased to hear this.
